# Specific (sialyl-)Lewis core 2 *O*-glycans differentiate colorectal cancer from healthy colon epithelium

**DOI:** 10.7150/thno.72818

**Published:** 2022-05-26

**Authors:** Katarina Madunić, Oleg A. Mayboroda, Tao Zhang, Julia Weber, Geert-Jan Boons, Hans Morreau, Ronald van Vlierberghe, Tom van Wezel, Guinevere S.M. Lageveen-Kammeijer, Manfred Wuhrer

**Affiliations:** 1 Leiden University Medical Center, Center for Proteomics and Metabolomics, Postbus 9600, 2300 RC Leiden, The Netherlands; 2 Leiden University Medical Center, Department of Surgery, Postbus 9600, 2300 RC Leiden, The Netherlands; 3 Leiden University Medical Center, Department of Pathology, Postbus 9600, 2300 RC Leiden, The Netherlands; 4 Department of Chemical Biology and Drug Discovery, Utrecht University, Universiteitsweg 99, 3584 CG Utrecht, The Netherlands

**Keywords:** Tumor associated carbohydrate antigens (TACAs), mass spectrometry, glycomics, therapeutic target

## Abstract

Cells are covered with a dense layer of carbohydrates, some of which are solely present on neoplastic cells. The so-called tumor-associated carbohydrate antigens (TACAs) are increasingly recognized as promising targets for immunotherapy. These carbohydrates differ from those of the surrounding non-cancerous tissues and contribute to the malignant phenotype of the cancer cells by promoting proliferation, metastasis, and immunosuppression. However, due to tumor tissue heterogeneity and technological limitations, TACAs are insufficiently explored.

**Methods:** A workflow was established to decode the colorectal cancer (CRC)-associated *O*-linked glycans from approximately 20,000 cell extracts. Extracts were obtained through laser capture microdissection of formalin fixed paraffin embedded tissues of both primary tumors and metastatic sites, and compared to healthy colon mucosa from the same patients. The released *O*-glycans were analyzed by porous graphitized carbon liquid chromatography-tandem mass spectrometry in negative ion mode.

**Results:** Distinctive *O*-glycosylation features were found in cancerous, stromal and normal colon mucosal regions. Over 100 *O*-linked glycans were detected in cancerous regions with absence in normal mucosa. From those, six core 2 *O*-glycans were exclusively found in more than 33% of the cancers, carrying the terminal (sialyl-)Lewis^X/A^ antigen. Moreover, two *O*-glycans were present in 72% of the analyzed cancers and 94% of the investigated cancers expressed at least one of these two *O*-glycans. In contrast, normal colon mucosa predominantly expressed core 3 *O*-glycans, carrying α2-6-linked sialylation, (sulfo-)Lewis^X/A^ and Sda antigens.

**Conclusion:** In this study, we present a novel panel of highly specific TACAs, based upon differences in the glycomic profiles between CRC and healthy colon mucosa. These TACAs are promising new targets for development of innovative cancer immune target therapies and lay the foundation for the targeted treatment of CRC.

## Introduction

Colorectal cancer (CRC) is one of the most common malignancies with over 900,000 deaths worldwide in 2020 1. Conventional therapeutic strategies for CRC include chemotherapy, radiation therapy, and surgery. However, despite the recently introduced and highly successful pre-symptomatic population screening, many CRC cases are still detected at an advanced stage, leading to unsuccessful treatment 2. In the past decade, new CRC therapies have emerged, such as specific inhibitor or antibody targeting of soluble proteins and cellular receptors 3. These include the biologics anti-VEGF (bevacizumab), anti-EGFR (cetuximab and panitumumab), anti-PD-1 immune checkpoint inhibitors (pembrolizumab, nivolumab) and BRAF V600E inhibitors (several combinations of vemurafenib, irinotecan, and cetuximab or panitumumab) 4, with many more in development. Unfortunately, these treatments are only efficient for a limited subset of patients, as many develop resistance to treatment 5. Therefore, the identification of new specific molecular targets and the development of new targeted therapies for CRC is essential for providing better treatment to a larger group of patients.

Various studies demonstrated altered glycosylation patterns of proteins and lipids as a hallmark of cancer 6,7. Changes in glycosylation originate from a shift in the expression of glycosyltransferases, modifications of their enzymatic activity, mislocalization in the endoplasmic reticulum or Golgi apparatus, availability of substrates or nucleotide sugar donors as well as competition between the enzymes 6. Characteristic glycan alterations in cancer include specific aberrant expression of incomplete carbohydrate structures or *de novo* expression of carbohydrate antigens also known as tumor-associated carbohydrate antigens (TACAs) 8. Of note, aberrant cancer glycosylation has already found its way into the clinics in form of the CA19-9 serological marker for *e.g.* pancreatic cancer and CRC, known as the sialyl-Lewis A (sLe^A^) carbohydrate antigen 9.

Changes in the expression of heavily glycosylated intestinal mucins were shown in CRC. More specifically, the expression of MUC2 and MUC4 is downregulated in CRC, whereas MUC1, MUC5AC and MUC17 are overexpressed 10, showing reduced expression of core 3 and core 4 structures with increased expression of *O*-linked truncated core 1 glycans, (sialyl-)T antigen, (sialyl-)Tn, as well as sialyl-Lewis X (sLe^X^) antigen 7. Several of these motifs are being evaluated as targets of immunotherapeutic monoclonal antibodies 11, despite their often limited specificity.

While the presence of these carbohydrate motifs in tumors is well-known, information on the underlying TACA structures is vastly lacking. The majority of these studies rely on detecting abnormal expression of glycosyltransferases in cancer and detection of terminal epitopes on all glycan types. No differentiation is made between the different glycan classes, such as *N*- and *O*-linked glycoproteins as well as glycolipids, and nonspecific TACA structures could be revealed 12-18.

In recent years, sensitive mass spectrometry (MS) based glycomics workflows were developed to enable untargeted screening of potential glycan markers derived from complex biological samples. However, tumor tissue heterogeneity is a major obstacle for cancer glycomics studies. The tumor microenvironment is populated by non-neoplastic cells such as immune cells or fibroblasts, which can hamper the detection of specific cancer associated glycan species. This issue can be largely overcome by sampling areas of interest with laser capture microdissection (LCM) prior to glycan release, as demonstrated in hepatocellular carcinoma by Hinneburg *et al.* 19,20.

In this study, we focused on decoding the tumor specific *O*-glycan signatures of CRC. The *O*-glycome was determined from both epithelial regions of primary tumors, metastatic sites and healthy colon tissue from the same patients. Furthermore, we established a high throughput workflow that sequentially releases the *N-*and *O-*glycans from LCM formalin fixed paraffin embedded (FFPE) tissues, followed by analysis using porous graphitized carbon liquid chromatography (PGC-LC)-MS/MS in negative ion mode, enabling a powerful separation of isomeric species as well as in-depth structural characterization of TACAs. This approach allowed us to identify CRC specific TACAs as potential targets of innovative cancer immunotherapy such as anti-TACA antibodies, adoptive T-cell therapies and antibody-drug conjugates.

## Results and Discussion

### A high throughput workflow for glycan analysis from laser capture microdissected colon tissues

First a high throughput workflow was established enabling the characterization of *O*-glycans from LCM FFPE tissue slides (**Figure S1**). This workflow is based on previously established protocols for glycan analysis from complex biological samples 21,22 as well as a formerly developed glycomic workflows using FFPE tissue sections 19. Briefly, specific regions of the FFPE tissue sections were extracted by LCM, such as normal colon mucosa (**Figure S2A**-**B**)**,** cancer epithelium (**Figure S2C-D**) and cancer stroma. Tissue lysates (20,000 - 25,000 cells) were used for protein immobilization on PVDF membrane plate wells. The *N*-glycans were released prior to *O*-glycan release to avoid interference. *O-*glycans were released from the immobilized proteins, purified and analyzed by PGC-LC-MS/MS. To ensure protein solubilization from formalin fixed material and binding of the solubilized proteins to the PVDF membrane, the amount of detergent used for the lysis was decreased to 1%, compared to the 4% used in the original FFPE tissue glycomics approach 19. This way the tedious chloroform/methanol protein precipitation step is avoided, as it resulted in irreproducible protein yields for small sample amounts. Regarding *O*-glycan detection, we used isopropanol enriched nitrogen gas to increase sensitivity and the quality of the MS/MS spectra, as described previously 22. Good precision was observed for the complete workflow and analysis, as depicted by the technical replicates as well as pooled samples in **Figure S3**, respectively.

### Glycosylation signatures in colorectal cancer

The presence of TACAs was investigated using paired primary tumors (T1-T12) and normal colon (C1-C12) from 12 CRC patients. Moreover, metastatic CRCs from liver metastases of six additional patients (M15-M21) were analyzed. Clinical data related to the samples used in our cohort is available in **Table S1** and** S2**. From the 12 cancer samples, eight were adenocarcinoma (AC), one was neuroendocrine carcinoma (NEC), and two were mucinous adenocarcinoma (MUC), of which one was partially signet-ring cell adenocarcinoma (T2). During the microdissection of the epithelial tumor regions and mucosal regions from normal colon, it was ensured that the regions were well separated from the surrounding submucosal and muscle layers of the colon and tumor stroma. In addition, the stromal regions for a subset of cancers with high stromal content were collected. *O-*glycosylation profiles were obtained for those regions separately.

**Figure 1:**
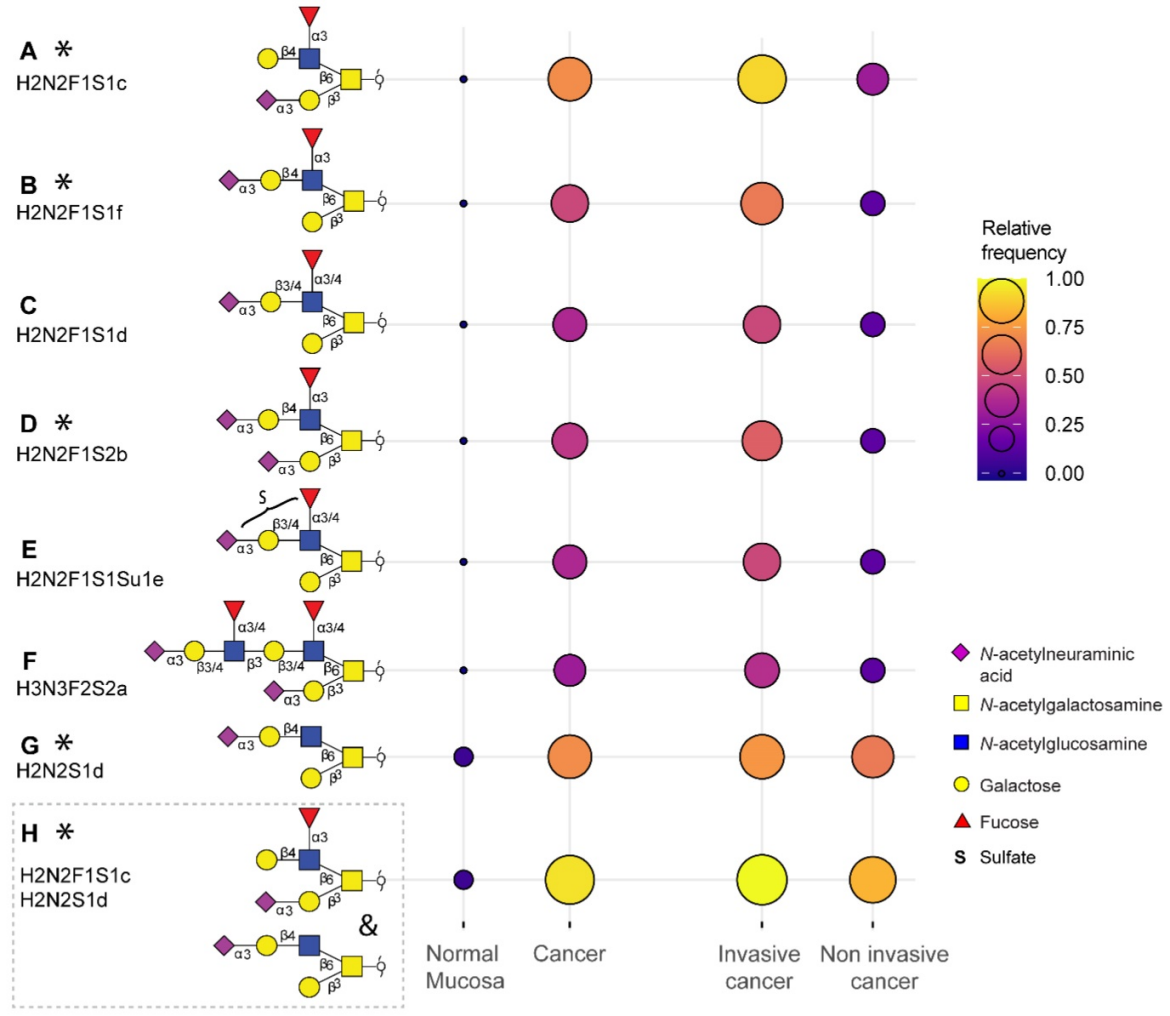
** Tumor associated carbohydrate antigens identified in colorectal cancer.** A subset of the O-glycans overexpressed in cancer which is detected in more than 33% of the cancers and undetectable in normal colon mucosa samples **(A, B, C, D, E, F)** or detected in maximum one normal mucosa **(G)**. Either one of the two TACAs in **(H)** is detected in 94% of the cancers. The selected TACAs show higher specificity for invasive cancer (Dukes stages C and D, n = 12) than non-invasive cancer (Dukes stages A and B, n = 6) (right panel). Structures confirmed by synthesized standards (J. Weber *et al.,* manuscript in preparation, 2022) are labeled with an asterisk. Blue square: *N*-acetylglucosamine, yellow square: *N*-acetylgalactosamine, yellow circle: galactose, red triangle: deoxyhexose, pink diamond: *N*-acetylneuraminic acid, S: sulfate modification.

**Figure 2:**
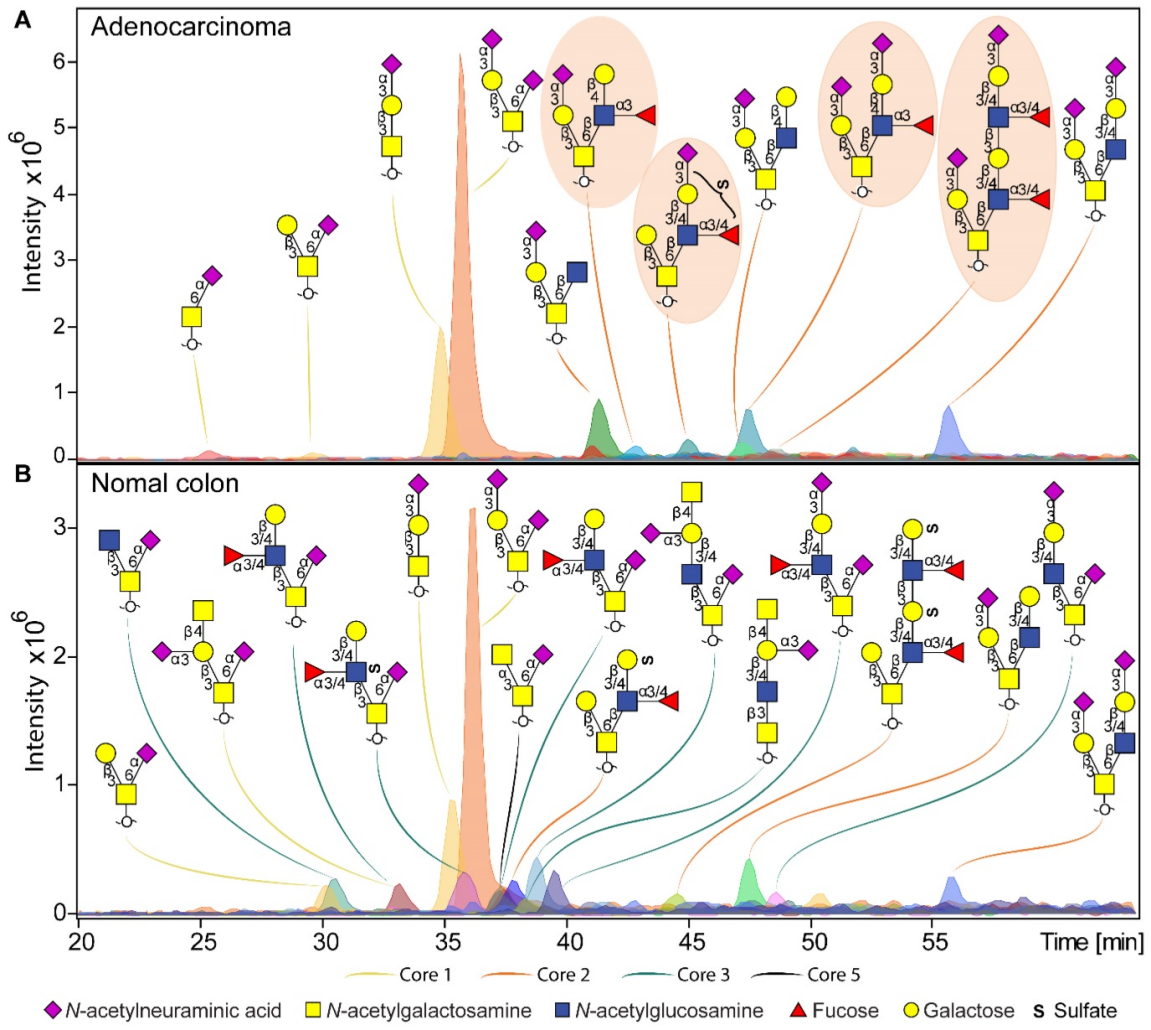
** Example of *O*-glycan chromatographic profile from (a) adenocarcinoma and (b) normal colon mucosa from the same patient (T8 vs C8). A)** The adenocarcinoma from patient 8 is characterized by specific expression of core 2 *O*-glycans, carrying terminal Le^X^ and sialyl-Lewis^X/A^ antigens, or just terminal α2-3 sialylation linked to the galactose. Presence of TACAs (**Figure 1**) are circled with orange background.** B)** Normal colon mucosa from the same patient shows expression of a diversity of core 3 structures, in most cases carrying an α2-6 linked sialic acid linked to the core GalNAc. Core 3 *O*-glycans are carrying terminal Sda antigens, as well as Le^X/A^ and sulfo-Le^X/A^ epitopes. The TACAs observed in adenocarinoma are not detected in the normal colon mucosa from the same patient. Blue square: *N*-acetylglucosamine, yellow square: *N*-acetylgalactosamine, yellow circle: galactose, red triangle: deoxyhexose, pink diamond: *N*-acetylneuraminic acid, S: sulfate modification.

**Figure 3:**
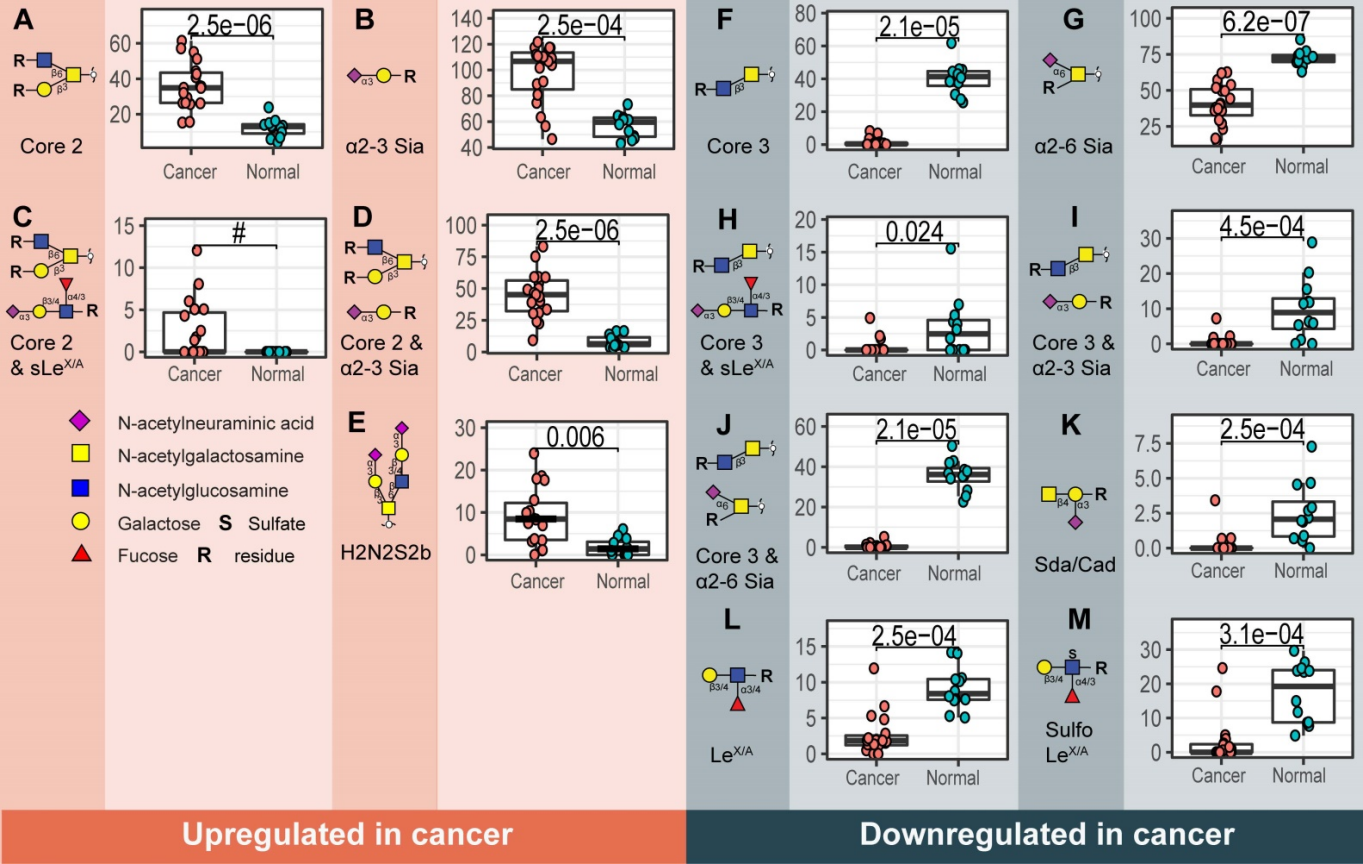
** Structural *O*-glycan features that differentiate between cancer and normal colon mucosa.** Statistically significant upregulation in cancer is found for various *O*-glycan features; **A)** Core 2 *O*-glycans, **B)** α2-3 sialylation, **C)** core 2 *O*-glycans with sLe^X/A^ and **D)** core 2 *O*-glycans with terminal α2-3 sialylation linked to a galactose together with **E)** one individual *O*-glycan with composition H2N2S2. Whereas, **F)** core 3 *O*-glycans, **G)** α2-6 sialylation of the core GalNAc, **H**) core 3 *O*-glycans with sLe^X/A^ antigen,** I)** core 3 *O*-glycans with terminal α2-3 sialylation, **J)** core 3 *O*-glycans with α2-6 sialylation, **K)** Sda antigen, **L)** Le^X/A^ and **M)** sulfo-Le^X/A^ antigen show statistically significant downregulation in cancer. Differences between groups were tested using Wilcoxon-Mann-Whitney non-parametric statistical test. Correction for multiple testing was made using the Benjamini-Hochberg method. ^#^ no p-value is reported as there is no variance in the control group. Blue square: *N*-acetylglucosamine, yellow square: *N*-acetylgalactosamine, yellow circle: galactose, red triangle: deoxyhexose, pink diamond: *N*-acetylneuraminic acid, S: sulfate modification.

Overall, a total of 172 *O*-glycans were detected in the analyzed tissues (**Table S3** and** S4**). From those, 108 were observed in the cancer and stroma, with no expression in normal colon mucosa (**Table S5**)**.** From those, 100 *O*-glycans were solely detected in cancer (TACAs), whereas only 20 *O*-glycans were shared between cancer and normal colon mucosa (**Figure S4**). The majority of the TACAs have a core 2 structure carrying sialyl-Lewis^X/A^ (sLe^X/A^) antigens or an α2-3-linked sialic acid attached to the galactose (Gal) (**Figure S4, S5, S6B** and** S7**). Six core 2* O*-glycans that were not detected in normal mucosa, but exclusively found in more than six cancers (relative frequency > 33%) are depicted in **Figure 1A-F** and listed in **Table S6** and** S7**. One core 2 *O*-glycan, with terminal α2-3 sialylation of Gal on the 6-arm, is found in one normal mucosa sample and in 72% of the cancers (**Figure 1G**). A sialylated core 2* O*-glycan with terminal Le^X/A^ antigen (**Figure 1A**) was absent from normal mucosa and found in 72% of the cancers. In particular, at least one of the two TACAs depicted in **Figure 1H** were detected in 94% of the cancers investigated in our study. Interestingly, the only sample in which those *O*-glycans were not detected was a poorly differentiated adenocarcinoma, specifically classified as solid type without glandular formation, which is usually associated with better patient prognosis 23. MS/MS spectra of the selected TACAs are shown in **Figure S8.** Additional confirmation was found by performing standard addition experiments with available synthesized standards as exemplified in **Figure S9.** An example of TACA expression is illustrated in **Figure 2A**, which shows the expression of four specific TACAs in the adenocarcinoma tissue from patient 8, while those *O*-glycans were not detected in the normal colon mucosa from the same patient (**Figure 2B**). This pattern of TACA expression was consistently observed across the entire dataset in cancer regions but not in presumably unaffected epithelium of the same patient. Therefore, our data provides strong evidence for existence of highly specific *O*-glycans which are not present in the normal colon mucosa tissue. Moreover, significantly different *O*-glycomic traits (summarized in **Figure S5**) were found between CRC and normal mucosa (**Figure 3**; **Table S8**, **S9** and **S10**). While core 2 *O*-glycans are overexpressed, core 3 *O*-glycans are downregulated in cancer (**Figure 3A** and** F**). A different expression is observed for sialylation, where terminal α2-3 sialylation is overexpressed and α2-6 sialylation is downregulated in cancer (**Figure 3B** and** G**). Moreover, a core 2 *O*-glycan with terminal α2-3 sialylation of Gal is detected in the normal colon mucosa (**Figure 3E**), and is strongly overexpressed in cancer. Differences were also found regarding antigen expression, namely terminal sLe^X/A^ and terminal α2-3 sialylation were upregulated in cancer in the context of core 2 *O*-glycans (**Figure 3C** and **D; Figure S7** and** S10A**). Although the presence of sLe^X/A^ was associated with several epithelial cancers 24-27, no specific targets were previously identified. Notably, the sLe^X/A^ antigens and terminal α2-3 sialylation were also carried by core 3 structures in normal colon mucosa and mucinous adenocarcinomas but showed a downregulation in cancer (**Figure 3H** and **I; Figure S10B** and **Table S10**). *O*-glycans carrying this antigen but with a core 4 structure did not show a difference between cancer and normal mucosa (**Figure S10C** and **Table S10**). Based on our results, we suggest that the specificity of the immunotherapeutic antibodies should move towards TACAs with specific (s)Le^X/A^ epitopes carried by core 2 *O*-glycans, and not those that are present on core 3 or core 4. Interestingly, a previous study evaluated the influence of glycan cores for specificity of sLe^X^ antibodies, and revealed that a commonly used KM93 antibody binds to sLe^X^ on core 2 *O*-glycans, and not on core 3 *O*-glycans 28. Moreover, the monoclonal antibody CHO131, which targets sLe^X^, demonstrated partial specificity for core 2 *O*-glycans 29, revealing high expression of these glycans in primary CRC and liver derived metastatic carcinoma 30. Core 2 sLe^X/A^ epitopes are recognized as cancer virulence factors as they bind selectin ligands and mediate cancer metastasis 31-33. Their expression was associated with less differentiated areas of the tumors with glandular formation 30,34. This reveals the potential for development of specific antibodies targeting a single core-type *O*-glycan in combination with cancer associated (s)Le^X/A^ epitopes.

### Glycosylation signatures in normal colon mucosa

In the normal colon mucosa nearly 80 *O*-glycans were detected and 26 of them were specific for the mucosa, belonging mainly to *O*-glycans with a core 3, carrying an α2-6 sialylation on the core GalNAc, terminal Sda antigen and (sulfo-)Le^X/A^ antigens (summarized in **Figure S5**). Most importantly, a statistically significant downregulation was found in cancer for these structures (**Figure 3 F-M**). A total of 12 *O*-glycans were shared between cancer, mucosa and stroma, which were mainly sialylated core 1 *O*-glycans, such as ubiquitous sialyl-3T, and disialyl-T (**Figure S7** and** S12**) and core 2 types with terminal α2-3 sialylation on Gal (**Figure 3E; Figure S4** and **S7**). Previous studies on the glycosylation of secreted mucus from normal colon mucosa reported expression of very similar glycosylation features, namely a high expression of core 3 structures carrying (sulfo-)Le^X/A^ and Sda antigens. Yet the abundance of sialylated core 1 *O*-glycans was lower than observed in our study 35,36. Potentially, the glycosylation of secreted mucins could differ from cell glycosylation. However, those signatures could also partially originate from the immune cell infiltrate within the healthy colon mucosa, which could not be fully excised using LCM.

### Glycosylation of specific cancer types

Stratifying the observed *O*-glycomic signatures by cancer type yielded further information (**Figure S11** and** S12**). No clear clustering was found between cancers with the same differentiation grade, tumor stroma ratio and location (**Figure S13** and** S14**). The tumors with high immune infiltration did not show a specific *O*-glycan signature (**Figure S14B**). Moreover, microsatellite instable (MSI) tumors, known for high immune infiltration, did not cluster together on the principal component analysis (PCA) plot (**Figure S13A**). However, several glycan features showed associations with MSI, tumor-stroma ratio and invasiveness (**Figure S15**). Namely, the MSI cancers were characterized by downregulation of α2-3 sialylation and core 1 *O*-glycans including disialyl-T and sialyl-3T, whereas sialyl-6T antigen was upregulated **(Figure S15A-E)**. Tumors with high tumor-stroma ratio (low stromal content) showed lower expression of core 1 *O*-glycans including disialyl-T antigen, with higher expression of core 2 *O*-glycans **(Figure S15F-H)**. While, sialyl-Tn antigen was detected in both cancer and healthy mucosa (**Figure S12**), it showed a trend towards upregulation in cancer. The highest expression was found in a poorly differentiated stage 4 MSI tumor (T11), two mucinous MSI adenocarcinomas (T2 and T3), and a neuroendocrine carcinoma (T5). Previous studies reported higher expression of sialyl-Tn in poorly differentiated and mucinous colon adenocarcinomas, but no associations with MSI were investigated 37. It is yet to be determined whether MSI status is associated with expression of specific glycosyltransferases leading to higher expression of α2-6-sialylated Tn and T antigens.

Mucinous adenocarcinomas (T2 and T3) expressed 43 specific *O*-glycans (**Figure S11**), mostly carrying sLe^X/A^ antigen, on core 2 but also core 4 *O*-glycans (**Figure S11** and** S12**). Both tumors were MSI, which are typically classed as low-grade cancers 38. Core 4 *O*-glycan expression can be explained by a strong downregulation of *ST6GALNAC1* and *3* in cancer, leading to the branching of the core 3 precursors in cancers with expression of core 4 synthase *GCNT3* 39. Neuroendocrine carcinoma (T5) showed a specifically high expression of Sd^a^ antigen, compared to other cancer types which displayed low to no expression (**Figure S12**).

Cancers with lymph node invasion (LNI) or invasion to distant organs, including Dukes stages C and D (metastasis adenocarcinoma), showed overexpression of sLe^X/A^ antigens on core 2 *O*-glycans, as well as α2-3 sialylation on core 2 *O*-glycans, compared to non-invasive cancers (Dukes stage B) (**Figure S15I-J**). Specifically, they presented with a higher expression of the selected TACAs on core 2 *O*-glycans compared to non-invasive cancers (**Figure 1; Table S6**). Due to their absence or limited expression in the normal colon mucosa, and high specificity for invasive cancer (**Figure 1**), these TACAs can serve as promising targets for treatment of invasive CRC.

Metastatic adenocarcinoma derived from liver metastases showed expression of 20 specific *O*-glycans, mainly core 2 *O*-glycans carrying terminal α2-3 sialylation (**Figure S11**). Two examples, M16 and M15, expressed mostly sLe^X/A^, Le^X/A^ antigens, whereas the rest was enriched with mostly truncated core 1 *O*-glycan signatures such as sialyl-3T, disialyl-T as well as core 2 α2-3 sialylated *O*-glycans (**Figure S12**).

Although our study is at the forefront of all currently available in-depth glycan characterization studies, it only included 18 patients. More insights into CRC subtype-specific glycan signatures might arise from follow-up studies using larger cohorts, including a better coverage of mucinous, neuroendocrine and metastatic carcinoma. Moreover, it would be of major clinical relevance to explore paired tissues of primary *versus* metastatic carcinomas to gain insights into specific signatures of metastatic CRC.

### Pathways of *O*-glycan biosynthesis in colorectal cancer

**Figure 4:**
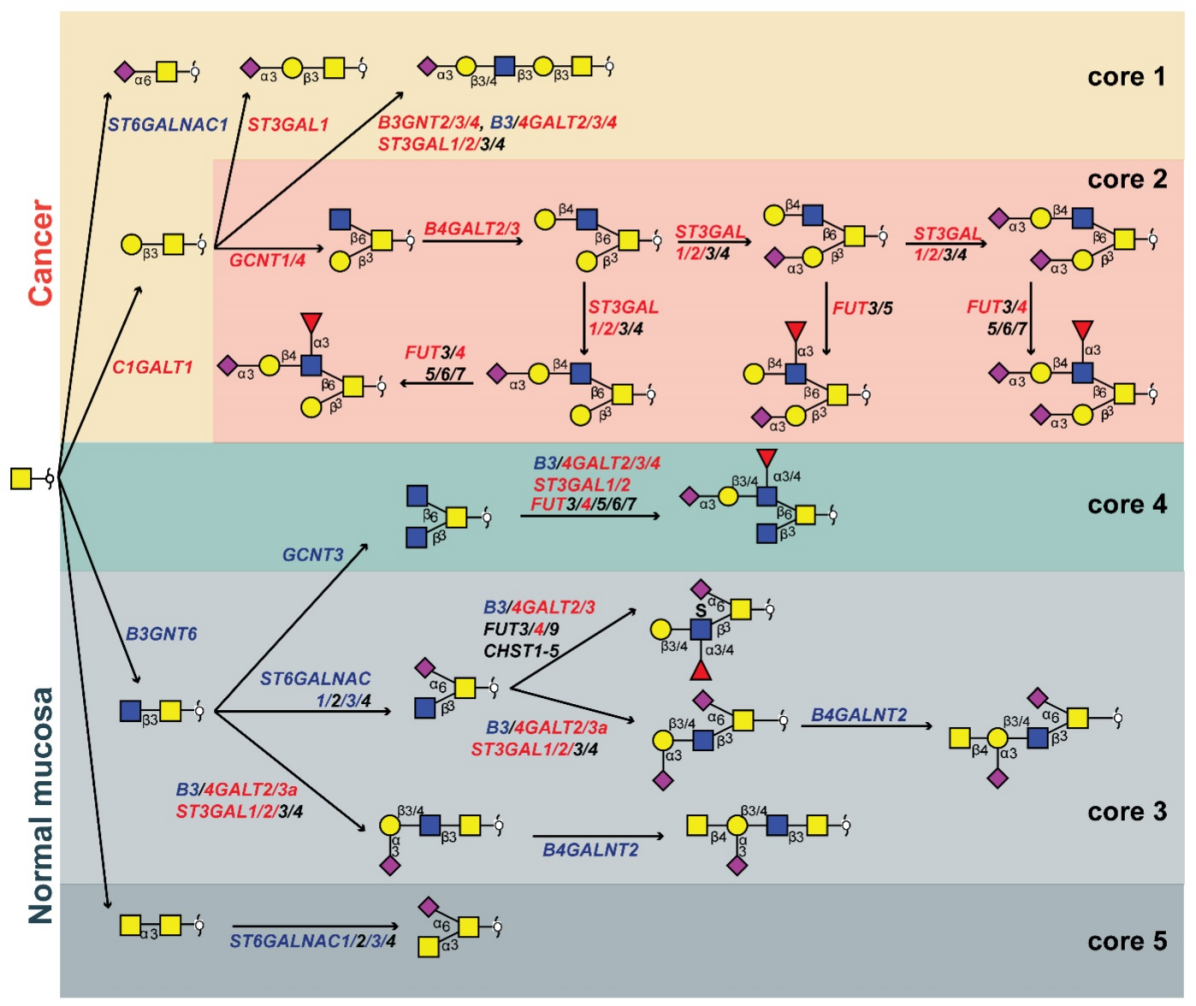
** Proposed biosynthetic model explaining the differences in glycosylation of colorectal cancer and normal colon mucosa.** The most abundant structures in cancer (upper red and yellow panel) and normal colon mucosa (lower green and gray panels) are depicted together with genes encoding for the GTs involved in their biosynthesis. The biosynthetic pathways of different core structures are labelled with different colors. Pathways upregulated in cancer are marked in orange and yellow, whereas pathways downregulated in cancer are marked in light and dark gray. Core 4 *O*-glycans show no statistically significant difference between normal mucosa and cancer, marked in green. Glycosyltransferase genes upregulated in cancer are labelled with red and those downregulated in cancer are displayed in blue. Blue square: *N*-acetylglucosamine, yellow square: *N*-acetylgalactosamine, yellow circle: galactose, red triangle: deoxyhexose, pink diamond: *N*-acetylneuraminic acid, S: sulfate modification.

We created a biosynthetic model (**Figure 4**) explaining the *O*-glycomic changes that occur in cancer compared to normal colon mucosa by integrating the *O*-glycomics results with publicly available gene expression datasets 40 (**Figure S16**). Downregulation of *B3GNT6*, a core 3 synthase which adds β1-3-linked GlcNAc to the core GalNAc, leads to downregulation of core 3 structures, whereas upregulation of *C1GALT1* (core 1 synthase) leads to the upregulation of core 1 structures in cancer. Previously, expression of core 3 synthase was associated with better patient prognosis in pancreatic 41, prostate 42 and colon cancer 43. Moreover, the upregulation of *C1GALT1* in cancer was previously described to enhance proliferation, migration, invasion, and sphere formation *in vitro* as well as tumor growth and metastatic potential of CRC cells *in vivo* 44*.* This competition between core 1 and core 3 synthase as well as core α2-6 sialylation in the context of sialyl-Tn was suggested previously in colon cancer cells 45. While we did not observe statistically significant upregulation in the expression of sialyl-Tn antigen in cancer although a trend was present. Notably, the detection of sialyl-Tn was previously only associated with secretions of well-differentiated regions of CRC 34. The main enzyme responsible for the biosynthesis of sialyl-Tn antigen, *ST6GALNAC1,* is downregulated in cancer, however, a higher expression of sialyl-Tn antigen can be due to a mutation in the *C1GALT1C1* gene encoding for a chaperone (Cosmc) required for the activity of core 1 synthase, which could lead to a blockage of the alternative pathway 46. Additionally, it was previously suggested that the observed increase of sialyl-Tn expression in CRC is caused by low sialyl-Tn acetylation in tumor *versus* high sialyl-Tn acetylation in normal colon mucosa 47. Due to the high pH conditions during our glycan release step, acetylation of sialic acids is completely removed, preventing detection of acetylation in our study. Therefore, it is possible that previously reported overexpression of sialyl-Tn antigen in CRC was biased by the detection method.

On the other hand, upregulation of *GCNT1* and *GCNT4* (both core 2 synthases that add a GlcNAc to the core GalNAc in the β1-6 position, creating the 6-arm), leads to overexpression of core 2 structures. Upregulation of *GCNT1* and *GCNT4* was observed in the microdissected CRC tumors, but not in the The Cancer Genome Atlas (TCGA) dataset (**Figure S16**). Since the upregulation is not particularly high, we hypothesize that elevated levels of core 2 structures in CRC are mainly a consequence of the downregulation of the core 3 *O*-glycan biosynthetic pathway. Addition of a galactose residue to the 6-arm of a core 2 precursor by activity of β1-4-galactosyltransferases *B4GALT2* and *B4GALT3* (**Figure S16**) is upregulated in cancer 48,49, leading to the biosynthesis of a pentasaccharide with α2-3-linked sialic acid (**Figure 1G**). This is a TACA with high specificity for cancer and a precursor for other highly specific TACAs (**Figure 1B-E**). Moreover, the addition of another LacNAc creates a sialyl-dimeric Le^X/A^ antigen *O*-glycan which is also specifically found in cancer (**Figure 1F**). The fucosyltransferase *FUT4* involved in the biosynthesis of sLe^X^ antigen is overexpressed in colon cancer (**Figure S16**) and it was previously associated with lung adenocarcinoma metastasis and poor patient prognosis 50,51. It was also previously described that the upregulation of sLe^X/A^ antigens in CRC is due to downregulation of *B4GALNT2.* This adds β1-4-linked GalNAc creating the Sda antigen in the normal colon mucosa 52,53. This enzyme shows a downregulation in TCGA dataset, and only a trend was observed supporting the LCM dataset (**Figure S16**). Nevertheless, expression of *B4GALNT2* was previously described as a strong predictor of good prognosis in CRC as patients with high *B4GALNT2* expression displayed longer overall survival 53,54. Additionally, the expression of 6-sulfo-Le^X^ and sialyl-6-sulfo-Le^X^ was previously associated with normal colon epithelia, divergent from the expression of sLe^X^ in CRC 13. Furthermore, a lower expression of Le^A^ type antigens as well as sLe^A^ was found in CRC compared to normal colon mucosa due to a downregulation of β1-3-galactosyltransferase *B3GALT5* 55. Notably, it was demonstrated that the loss of acetylation is also the source of increase of sLe^X/A^ expression in CRC, due to the lack of antibody reactivity towards the acetylated antigen 56-58. Taken together, we propose that sLe^X/A^ antigens are present in both normal colon and cancer, in the context of different core structures and acetylation variants.

The elongation of core 1 structures is in competition with the core 2 biosynthetic pathway and the biosynthesis of sialyl-3T antigen (H1N1S1b). While this antigen is also detected in normal mucosa it is overexpressed in cancer (**Figure S12**), due to an upregulation of *ST3GAL1* (**Figure S16**) which was previously associated with lymph node invasion in CRC 59. The overall increase in α2-3 sialylation could be a consequence of the increased expression of both *ST3GAL1* and *ST3GAL2* observed in CRC (**Figure S16**). Recently, it was demonstrated that *ST3GAL2* knock-down causes a significant decrease in tumor cell proliferation and cell migration *in vitro*, as well as reduction of tumor invasiveness *in vivo* 60*.* Moreover, although not present in the LCM dataset, the downregulation of *ST6GALNAC3* is likely to contribute to the downregulation of α2-6 sialylation in CRC together with the upregulation of core 2 branching (**Figure S16**).

Lastly, we detected a core 5 structure (H1N2S1b) in the normal colon mucosa, which was described previously 36. With our PGC-LC-MS/MS approach we could confidently distinguish only one core 5 *O*-glycan (N2S1b) from isomeric core 3 *O*-glycans, as this glycan was previously characterized by NMR from bovine submaxillary mucin 61. However, it is unknown whether any of the remaining core 3 isomers originate from core 5.

In summary, we propose that the downregulation of core 3 synthase (*B3GNT6*) in cancer leads to the overexpression of branched *O*-glycans by the action of core 2 synthases *GCNT1* and *GCNT4.* The addition of a β1-4 galactose and α2-3 sialic acid on the 6-arm of the *O*-glycans forms a specific pathway in cancer, starting from a sialylated precursor (**Figure 1G**) leading to biosynthesis of fucosylated TACAs carrying terminal sLe^X^ antigen by the addition of a α1-3-linked fucose by *FUT4* overexpressed in cancer.

### Future perspectives

Analysis of specific molecular signatures from patient derived material is fundamental for understanding molecular mechanisms of disease onset and progression. As FFPE tissue sectioning is part of standard care in pathology, many tissues are widely available and remain well preserved at room temperature for a long period of time. Yet, tissue heterogeneity poses a problem for glycomic and transcriptomic analysis as it masks cell specific signatures. Dissecting specific regions of the tissue by LCM, largely overcomes this issue and enables analysis of glycomic signatures from specific cells of interest 62. We succeeded in the in-depth *O*-glycome analysis from a limited amount (20,000 - 25,000) of FFPE tissue-derived cells, which opens up the potential to use this workflow for future glyco-biomarker discovery in other types of tumors. This is a considerably higher number of cells compared to a previous approach reporting glycomics analysis from 1,000 cells 19. However, hepatocellular carcinoma cells are significantly bigger than colon cells. Moreover, the diversity of *O*-glycans found in the colon is much higher than in the liver, and this heterogeneity requires higher analytical sensitivity. Nevertheless, further improvements regarding the sensitivity of our current MS methods are essential, making single cell glycomics analysis possible to avoid any contamination from different cell populations. Moreover, the lack of retention of monosaccharides, and the limited retention of neutral disaccharides on PGC material, precluded or severely hindered the analysis of Tn and T antigens, respectively. Recently, a C18 nanoLC-MS based workflow for analysis of reducing end-labeled *O*-glycans overcomes this obstacle, and could be used as a complementary method to PGC-LC-MS in future experiments 63.

Determining the specificity of different glycosyltransferases for core structures, differing arms of branched glycans as well as expression of TACAs on different glycan classes, is of great importance. Targeting glycans may have several benefits compared to protein targeting. Namely, the TACAs are expressed on the cell surface, directly accessible to therapeutics and can be carried by multiple proteins, reflecting the overall glycosylation phenotype of the cell, providing a broader tumor targeting strategy 64. Ideally, the expression of TACAs is absent or limited in normal colon mucosa. However, some of the TACAs (H2N2F1S1c and H2N2F1S2b) have been reported as selectin ligands on P-selectin glycoprotein ligand-1 (PSGL-1) in HL-60 cells, important for the leukocyte extravasation through the endothelium, albeit only at very low relative abundances 65,66. Cancer cells carrying the same ligands may employ this physiological mechanism for successful metastasis to distant organs, which is why it is important to target these antigens, particularly in invasive carcinoma 24. Most human tissues show absent or low expression of *O*-glycans with compositions H2N2F1S1, H2N2F1S2, and H2N2S1 (see glycan structure database for human tissue on: *www.functionalglycomics.org*), although no direct link can be made, since the previously used methodology only offers glycan composition analysis. Low expression levels were reported in the human colon (H2N2S1) jejunum (H2N2S1, H2N2F1S1), heart (H2N2S1, H2N2F1S2) liver (H2N2S1, H2N2F1S2), spleen (H2N2S1) and lung (H2N2S1, H2N2F1S1) tissue. A more in-depth study comparing hepatocellular carcinoma and healthy liver tissue, performed on a limited set of samples by PGC-LC-MS, did show a low expression of *O*-glycans with H2N2F1S1 composition in normal liver tissue with a significantly increased expression in hepatocellular carcinoma 19. A detailed study of *O*-glycomic signatures in a small gastric tissue set showed expression of H2N2F1S1c in one gastric tumor, one tumor adjacent and one normal gastric mucosa tissue 67. Analysis of human skin revealed expression of H2N2S1d, without expression of fucosylated TACAs 68. In summary, compositional analysis by MS revealed potential expression of TACAs in other normal human tissues. However, this needs to be validated using isomer specific methodologies such as PGC-LC-MS, using synthesized standards. Alternatively, development of core glycan specific monoclonal antibodies will aid higher throughput detection of the TACAs, and provide insights into their expression in specific cells of the same tumors. The detection of the TACAs will not be hindered by abundant acetylation, as mentioned previously, since CRC tumors are characterized by loss of acetylation compared to normal colon 56.

While there is no doubt that the differences in the TACAs patterns between the cancer and healthy mucosa are a consequence of changes in the glycosylation machinery of the cells, it remains unclear whether the TACAs are protein specific, and whether the differences in their expression are related to mucin expression. With the aim to increase immunogenicity and specificity of glycan targets, TACAs are often coupled to protein carriers, either adjuvants or pathological carriers such as mucins. Therefore, it will be important to determine the protein carriers of TACAs. Previously, aberrant expression of Tn antigen was found on MUC1 membrane glycoprotein, and anti-Tn and T antigen expressed on MUC1 showed efficiency in a phase I clinical trial on ovarian, breast and cervical cancer 69 but no effect on patient outcome in a phase II clinical trial 70. Another monoclonal antibody targeting Tn-antigen on MUC1 showed promising preclinical results for targeting breast cancer; however the results need further validation 71. Additionally, genetically modified T-cells expressing chimeric antigen receptors (CAR-s) targeting Tn glycoforms of MUC1 showed promising cytotoxicity in xenograft models of T-cell leukemia and pancreatic cancer 72. Our study showed that core 2 sialylated or core 2 (s)Le^X/A^ carrying *O*-glycans have the highest specificity for CRC, however, more research is needed to determine if these signatures are mucin specific, and whether bispecific antibodies or CAR-T cells targeting both the protein carrier and the cancer specific glycans will increase the specificity and immunogenicity of the developed therapeutics.

## Conclusions

In this study, we present a novel panel of highly specific TACAs, based upon changes in the *O-*glycomic profiles between CRC and healthy colon mucosa. These TACAs are promising new targets for development of innovative cancer immunotherapies and lay the foundation for the treatment of invasive CRC.

## Methods

### Materials and reagents

Hematoxylin (cat. nr. GHS232), sodium dodecyl sulfate solution 20% (cat. nr. 05030), trifluoroacetic acid (TFA; cat. nr. 1.38178.0050), tris(hydroxymethyl)amino-methane (Tris; cat. nr. 252859; lot#BCBM2559V), sodium borohydride (cat. nr. 452882; lot nr. STBD8912V), hydrochloric acid (cat. nr. 258148; lot nr. SZBD3100V), DL-dithiothreitol (DTT; cat. nr. D0632; lot nr. SLBW0160), ammonium bicarbonate (cat. nr. 09830; lot nr. BCBQ6426V,) cation exchange resin Dowex 50W X8 (cat. nr. 217492; lot nr. MKCH2513), ammonium acetate (cat. nr. A1542) and polyvinylpyrrolidone mw 40,000 (PVP-40; lot.nr. BCBM0949V), 20% sodium dodecyl sulfate (SDS) solution in H_2_O (cat. nr. 05030-1L-F) were purchased from Sigma Aldrich (St. Louis, MO). Ethanol (EtOH; cat. nr. 100983.1000), NaCl (cat. nr. 1.06404.1000) and methanol (MeOH; cat. nr. 1.06009.2500) were purchased from Merck (Darmstadt, Germany). Acetonitrile LC-MS grade (cat. nr. 01203502) was obtained from Biosolve (Valkenswaard, the Netherlands). Glacial acetic acid (cat. nr. A6283; lot nr. SZBG2660H) and potassium hydroxide (cat. nr. P1767) were purchased from Honeywell Fluka (Charlotte, NC). PNGase F (*Flavobacterium meningosepticum* recombinant in *E. coli*; Cat No. 11365193001) was obtained from Roche (Mannheim, Germany). SPE bulk sorbent Carbograph (S*Pure Extract-Clean SPE Bulk Packing Material, 38-125µm, cat. nr. SPU-5122145) was acquired from BGB Analytik Benelux B.V. (Harderwijk, the Netherlands). MultiScreen® HTS 96-multiwell plates (pore size 0.45m) with high protein-binding membrane (hydrophobic Immobilon-P PVDF membrane) and 96-well PP Microplate (cat. nr. 651201; lot nr. E1708385) were purchased from Millipore (Amsterdam, the Netherlands), 96-well PP filter plate (cat. nr. OF1100) was obtained from Orochem technologies (Naperville, Il). MembraneSlide 1.0 PEN (cat.nr. 415190-9041-000) and Adhesive Cap 500 µL tubes (cat.nr. 415190-9211-000) were purchased from Carl Zeiss Microscopy (Göttingen, Germany). All buffers were prepared using Milli-Q water (mQ) generated from an ELGA system (Millipore, Amsterdam, the Netherlands. Chemically synthesized glycopeptide standards were provided by Utrecht University (Weber *et al.*; manuscript in preparation).

### FFPE tissue sectioning and staining

Paired primary tumor (T1-T12) and adjacent normal colon mucosa (C1-C12) from 12 patients were selected for the cohort. Additionally, 6 metastatic cancers obtained from liver metastases of a separate patient cohort were included (M15- M21). The FFPE blocks were sectioned (5 µm) using a microtome and mounted onto glass slides for hematoxylin and eosin (H&E) staining, or sectioned (10 µm) and mounted onto PEN membrane slides for LCM. All slides were dried overnight at 37 ˚C and stored at 4 ˚C.

Slides used for LCM were deparaffinized using xylene (three washes of 5 mins) and washed with absolute EtOH (two washes of 2 min). The slides were then rehydrated in a series of EtOH by submerging the slides in 85% EtOH, followed by 70% EtOH and distilled water. Hematoxylin was applied for 20 sec. The slides were then washed with demineralized water for two minutes and dehydrated with increasing EtOH series by submerging in 70% EtOH, followed by 85% EtOH and finally absolute EtOH. The slides were briefly air dried and stored at 4 ˚C. The slides used for pathologist annotation were H&E stained following the routine protocols.

### Pathologist annotation

An expert pathologist annotated tumor and healthy epithelial tissue regions from H&E stained slides by careful inspection under the microscope. Differentiation grade was determined by assessing glandular formation.

### Laser capture microdissection

Tissue sections mounted on PEN membrane slides for LCM were carefully inspected under the microscope. Target areas marked on H&E slides by the pathologist were located, and the laser was positioned accordingly (**Figure S2**). To ensure that comparable amounts of tissue were used for each sample, cells were counted in three different regions of the tissue in an area of approx. 2,500 µm^2^. An average of the count was used to extrapolate the area needed to be cut, in order to obtain samples containing approximately 20,000 cells. The large difference in cell size between hepatic and colon cells, compared to a previous approach 19, was taken into account by performing the LCM on 10 µm thick tissue sections, enabling the extraction of sufficient material for the analysis. LCM was performed using PALM RoboSoftware and collected in adhesive cap 500 µL tubes. Considering the healthy colon mucosa is surrounded by infiltrated lymphocytes which could not be separated from the epithelial cells successfully, we also extracted lymphoid follicles from different normal colon tissues and pooled them together to obtain a potentially confounding immune cell glycan profile (IM). Moreover, we extracted the stroma controls for the tumors with high stromal content which may be contaminated with cancer associated fibroblasts and immune cells (ST4, ST6, ST7, ST11, ST12, SM19, SM21).

### Glycan release and purification

Lysis buffer (100 µL) containing 100 mM Tris HCl, 0.1 M DTT, 100 mM NaCl, and 1% SDS was added to microdissected tissues collected in adhesive caps. The pieces were carefully collected in lysis buffer from the lid of the adhesive caps and transferred to 1.5 mL Eppendorf tubes. Prior to sonication using a Branson sonicator rod (three times for 15 sec, with output power 2/10) the tubes were placed on ice. During sonication, the samples were kept on ice and left 20 sec to cool down between each cycle. Furthermore, the samples were incubated at 99 ˚C for 60 min with mild agitation (400 rpm). After incubation, the samples were slowly cooled down at room temperature (RT). In the meantime, a PVDF membrane plate was preconditioned using 100 µL of 70% EtOH, until the membrane turned opaque, followed by an additional wash using 100 µL of MQ. Upon rewetting the membrane with 5 µL of 70% EtOH, 100 µL of mixed sample lysates were loaded onto the membrane wells. Additionally, a tissue lysate (containing approximately 8 x 10^4^ cells) was split into three separate samples (TECH1, TECH2, and TECH3) and processed independently (different wells of the PVDF membrane plate) to check for technical variability. The plate was incubated at RT on a shaker for 20 min to ensure binding. Unbound material was removed by centrifugation at 500 *x g* and washed with 100 µL of MQ. Then, 40 µL of 0.5% PVP-40 in MQ was added to the PVDF membrane wells to block the membrane and incubated for 5 min on a shaker. Upon removal of the blocking agent by centrifugation, the membrane wells were extensively washed, two times with 100 µL of PBS, two times with 100 µL of 10 mM ammonium bicarbonate followed by two times 100 µL of MQ. Each time the washing agent was removed by centrifugation at 500 *x g* for 1 min. Since the addition of PVP-40 increases the water flux through the membrane, the hydrophilicity is also increased 73. Therefore, to avoid enzyme mixture passing through, 10 µL of MQ was added to each well of the PVDF membrane plate to soak the membrane and fill the space underneath it. The plate was incubated on a shaker with light agitation for 5 min. The enzyme mixture (15 µL) containing 2U of *N-*glycosidase F was added to each well followed by an incubation of 15 min at 37 °C. Lastly, 15 µL of water was added on top, to prevent drying out the membrane overnight, and placed in the 37 ˚C incubator. The next steps were performed as previously described 22. Briefly, the following day, upon recovery of *N*-glycans, 50 µL of 0.5 M sodium borohydride in 50 mM potassium hydroxide solution was added to each well and incubated for 16 h at 50 ˚C. The samples were cleaned using self-packed cation exchange columns in 96-well plates, followed by PGC-SPE. The samples were dried and stored at -20 ˚C until analysis.

### PGC-LC-MS/MS measurements

Prior to analysis, the samples were resuspended in 15 µL of MQ and 1 µL of each sample was pooled together in a separate vial for a quality check and method optimization (QC). The QC pool and a bovine submaxillary mucin standard were used to check instrument performance each day of measurement. A total of 6 µL of each tissue sample was injected for analysis (40%). A custom-made trap column (size 30 × 0.32 mm) packed with 5 μm particle size PGC stationary phase was used to load the samples using 100% buffer A (10 mM ammonium bicarbonate) at a loading flow of 6 μL/min. The packing material was obtained from Hypercarb PGC analytical column (size 100 × 4.6 mm, 5 μm particle size, Thermo Fisher Scientific, Waltham, MA). The glycans were separated on a custom-made PGC column (100 mm × 75 μm, 3 μm particle size; packing material obtained from Thermo Fisher Scientific, Waltham, MA) at a 0.6 μL/min flow rate by applying a linear gradient from 1% to 50% of buffer B (60% acetonitrile, 10 mM ammonium bicarbonate) over 73 min. A constant column temperature of 45 ˚C was maintained. A part of the samples were remeasured to resolve isomers with a composition of H2N2F1S1 (isomer c and f). A different gradient was used for this purpose, ranging from 1% to 50% of buffer B over 110 minutes at 35 ˚C. The peak ratios of the resolved peaks were taken to extrapolate the peak areas from the original measurement (**Table S11**). The LC system was coupled to an amaZon ETD speed ESI ion trap MS using the CaptiveSpray™ source (Bruker Daltonics) with an applied capillary voltage of 1000 V in negative-ionization mode. The drying gas (N_2_) temperature was set at 280˚C and the flow to 3 L/min. The nebulizer gas pressure was kept at 3 psi enriched with isopropanol as described before 22. MS spectra were acquired in enhanced mode within a mass to charge ratio (*m/z*) range of 380-1850, target mass of smart parameter setting was set to* m/z* 900, ion charge control (ICC) to 40,000 and maximum acquisition time to 200 ms. MS/MS spectra were generated by collision-induced dissociation on the three most abundant precursors, applying an isolation width of 3 Th. The fragmentation cut-off was set to 27% with 100% fragmentation amplitude using the Enhanced SmartFrag option (30-120% in 32 ms) and ICC was set to 150,000.

### Data processing

Extracted ion chromatograms including all observed charge states (1^-^ and 2^-^) of the first three isotopes were used to integrate the area under the curve (AUC) for each individual glycan isomer using Compass DataAnalysis software (v.5.0). Peaks were manually picked and integrated. Relative quantitation was performed using the total area of all glycans within one sample as reference (100%). Glycan structures were identified by manual inspection of MS/MS spectra following known *O-*glycan biosynthetic pathways and available literature 61,74-77. In the structural interpretations all hexoses (H) are assumed to be galactose, all deoxyhexoses a fucose (F), all internal *N*-acetylhexosamines (N) an *N*-acetylglucosamine except for the N2S1b, confirmed to be core 5 by comparison with bovine submaxillary mucin glycans standard. All terminal *N*-acetylhexosamines attached to a galactose also substituted with a *N-*acetylneuraminic acid (S) were assumed to be *N*-acetylgalactosamine as part of the Sda/Cad antigen. All terminal *N*-acetylhexosamines attached to a galactose also substituted with a fucose were assumed to be *N*-acetylgalactosamine as part of blood group antigen A. Sulfate modification position was not determined due to partial sulfate migration, and no presence of diagnostic fragment ions was detected for the position on the Gal or GlcNAc residue. Therefore, its position in the glycan structures can be either on the Gal or the neighboring GlcNAc residues. Structures of the selected TACAs (**Figure 1A, B, D, G**) were confirmed by use of synthesized standards (J. Weber e*t al., manuscript in preparation,* 2022).

### Statistics

Data analysis and visualization were performed in ''R'' software using the following packages: tidyverse, readxl, caret, gridExtra, EnhancedVolcano, ggpubr, pcaMethods, Rcpm, ggrepel, tidyHeatmap, stringr and ComplexUpset. An imputation of the minimum positive value (0.0001) was performed to enable the use of PCA. Variables with near zero variance were excluded before computing the PCA model using nearZeroVar function from caret package. Differences between groups were tested using the Wilcoxon-Mann-Whitney non-parametric statistical test. P-values were adjusted for multiple testing using the “Benjamini-Hochberg” method.

### Gene expression datasets

TCGA transcriptomics data was downloaded via the firebrowse.org website. The data from laser capture microdissected CRC tissues with identifier GEO: GSE21815 was downloaded from https://www.ncbi.nlm.nih.gov/geo/query/acc.cgi?acc=GSE21815 40. Gene expression levels obtained from 132 microdissected CRC tumors were compared with microdissected normal colon epitheliums. Commercially available Human Whole Genome Oligo DNA Microarray Kit (Agilent Technologies) was used. Labeled cRNAs were fragmented and hybridized to an oligonucleotide microarray (Whole Human Genome 4×44K Agilent G4112F). Fluorescence intensities were determined with an Agilent DNA Microarray Scanner and were analyzed using G2567AA Feature Extraction Software Version A.7.5.1 (Agilent Technologies).

## Supplementary Material

Supplementary figures.

Supplementary tables.

## Abbreviations

AC: adenocarcinoma
AUC: area under the curve
CAR-s: chimeric antigen receptors
CRC: colorectal cancer
DTT: DL-dithiothreitol
EtOH: ethanol
F: deoxyhexose
FFPE: formalin fixed paraffin embedded
Gal: galactose
GalNAc: *N*-acetylgalactosamine
GalNAcol: reduced, innermost GalNAc
GlcNAc: *N*-acetylglucosamine
H: hexose
H&E: hematoxylin and eosin
ICC: ion charge control
LCM: laser capture microdissection
Le^X/A^: Lewis X/A
LNI: lymph node invasion
*m/z*: mass to charge ratio
MeOH: methanol
MS: mass spectrometry
MSI: microsatellite instable
MUC: mucinous adenocarcinoma
N: *N*-acetylhexosamine
NEC: neuroendocrine carcinoma
Neu5Ac: *N*-acetylneuraminic acid
PCA: principal component analysis
PGC-LC: porous graphitized carbon liquid chromatography
PSGL-1: P-selectin glycoprotein ligand-1
PVP-40: polyvinylpyrrolidone mw 40,000
rpm: rounds per minute
RT: room temperature
S: sialic acid
SDS: sodium dodecyl sulfate
sLe^A^: sialyl-Lewis A
sLe^X^: sialyl-Lewis X
Su: sulfate
TACAs: tumor associated carbohydrate antigens
TCGA: The Cancer Genome Atlas
TFA: trifluoroacetic acid
Tris: tris(hydroxymethyl)amino-methane
