# Supplementary material for: Specific (sialyl-)Lewis core 2 O-glycans differentiate colorectal cancer from healthy colon epithelium
Source: Theranostics. 2022 May 26;12(10):4498–512. doi: 10.7150/thno.72818 (PMC9254241; doi:10.7150/thno.72818)
Supplement: Supplementary file 1 — Supplementary figures. [file thnov12p4498s1.pdf]

Supplementary Information I

Specific (sialyl-)Lewis core 2 *O*-glycans differentiate colorectal cancer from healthy colon epithelium

Katarina Madunić[1], Oleg A. Mayboroda[1], Tao Zhang[1], Julia Weber[4], Geert-Jan Boons[4], Hans Morreau[3], Ronald van Vlierberghe[2], Tom van Wezel[3], Guinevere S.M. Lageveen-Kammeijer[1], Manfred Wuhrer[1*]

[1] *Leiden University Medical Center, Center for Proteomics and Metabolomics, Postbus 9600, 2300 RC Leiden, The Netherlands*
[2] *Leiden University Medical Center, Department of Surgery, Postbus 9600, 2300 RC Leiden, The Netherlands*
[3] *Leiden University Medical Center, Department of Pathology, Postbus 9600, 2300 RC Leiden, The Netherlands*
[4] *Department of Chemical Biology and Drug Discovery, Utrecht University, Universiteitsweg 99, 3584 CG Utrecht, The Netherlands*
* *Correspondence and requests for materials should be addressed to M. Wuhrer (email: m.wuhrer@lumc.nl)*

Table of Contents

Supplementary Information I 1
Figure S1 2
Figure S2 3
Figure S3 4
Figure S4 5
Figure S5 6
Figure S6 7
Figure S7 8
Figure S8 9
Figure S9 14
Figure S10 15
Figure S11 16
Figure S12 17
Figure S13 18
Figure S14 19
Figure S15 20
Figure S16 21

Figure S1

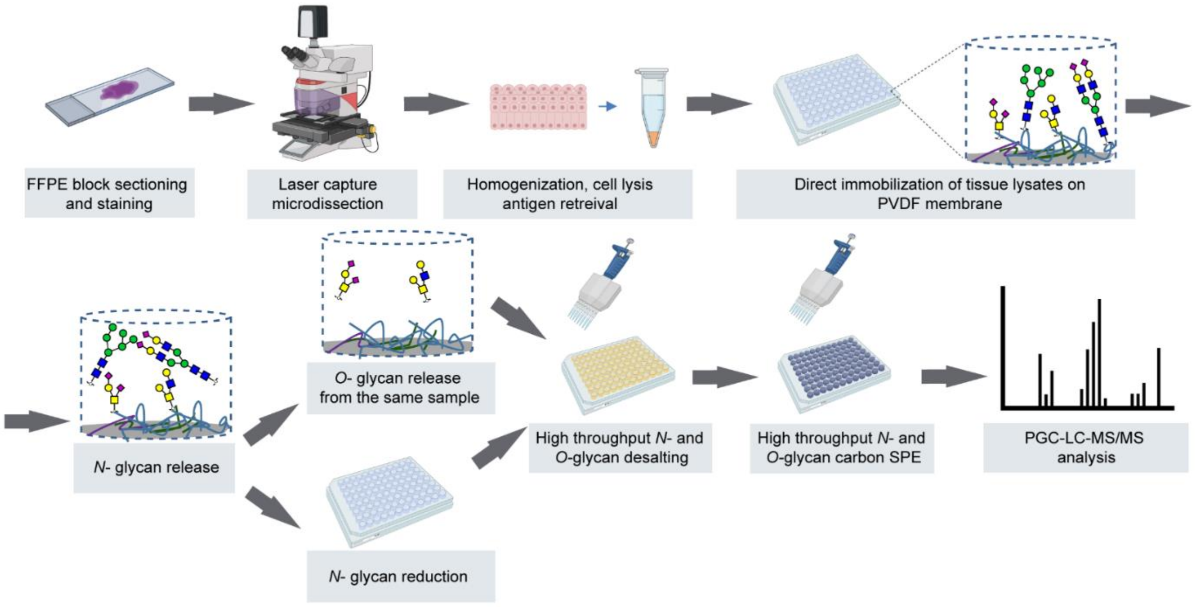

***Figure S1*. Schematic workflow for *N*- (optional) and *O*-glycomics from microdissected FFPE tissues.** Approximately 20,000 cells were extracted from tissues by laser capture microdissection and lysates were used for protein immobilization on PVDF membrane followed by an overnight *N*-glycan release by PNGase F digestion. After removal of the released *N*-glycans, *O*-glycans were released by reductive β-elimination, purified by cation exchange chromatography (CEX) and graphitized carbon solid phase extraction (SPE) packed in a 96-well filter plates and finally analyzed by PGC-LC-ESI-MS/MS. *Created with BioRender.com.*

Figure S2

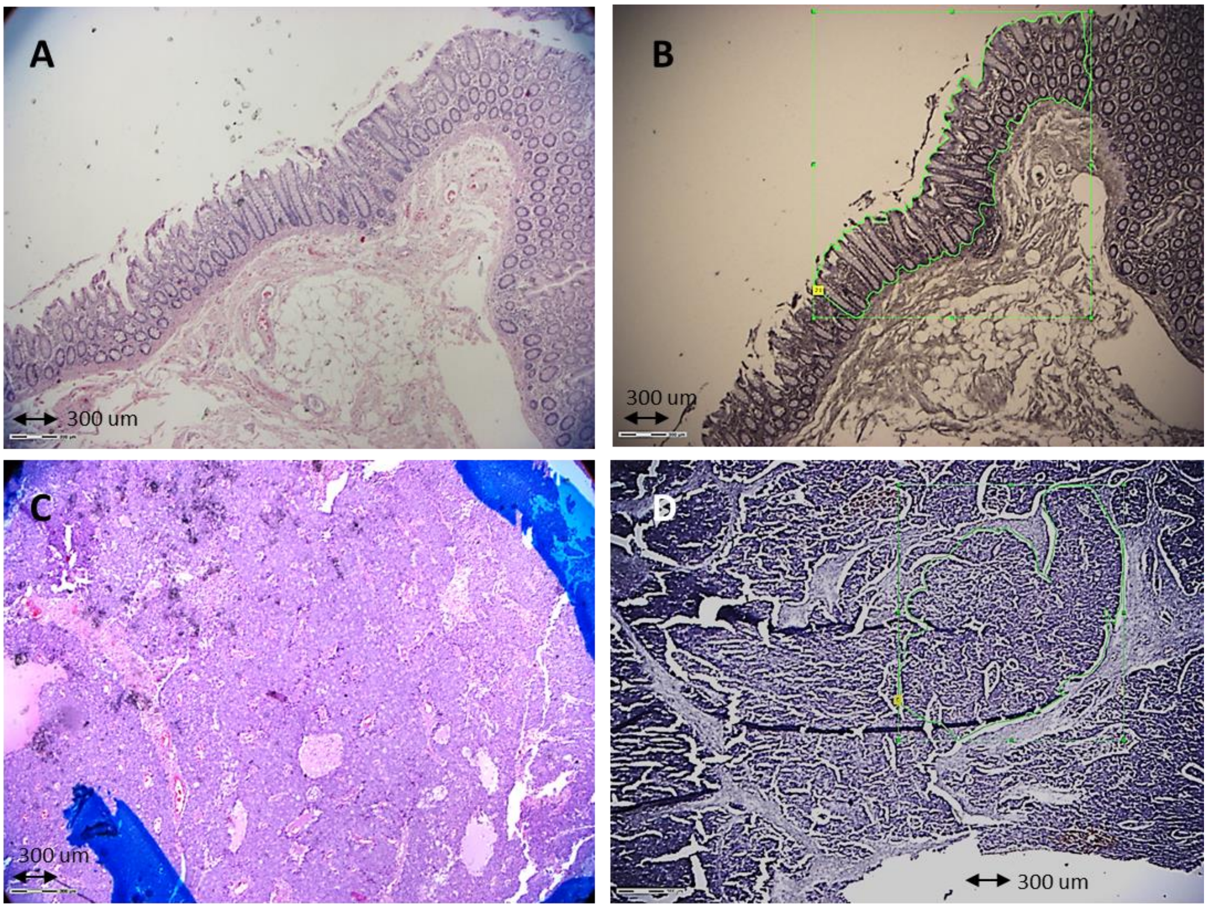

***Figure S2*. Representative images of FFPE specimens undergoing laser capture microdissection (LCM). A)** HE stained FFPE slide containing normal colon mucosa. **B)** Corresponding tissue slide used for laser capture microdissection with the target area outlined with green laser track marks. **C)** HE stained slide containing colon adenocarcinoma with **D)** corresponding tissue slide used for laser capture microdissection with the target area outlined with green laser track marks. More areas with similar size were dissected from the same tissue slide. Tumors were histologically characterized by a pathologist**.**

Figure S3

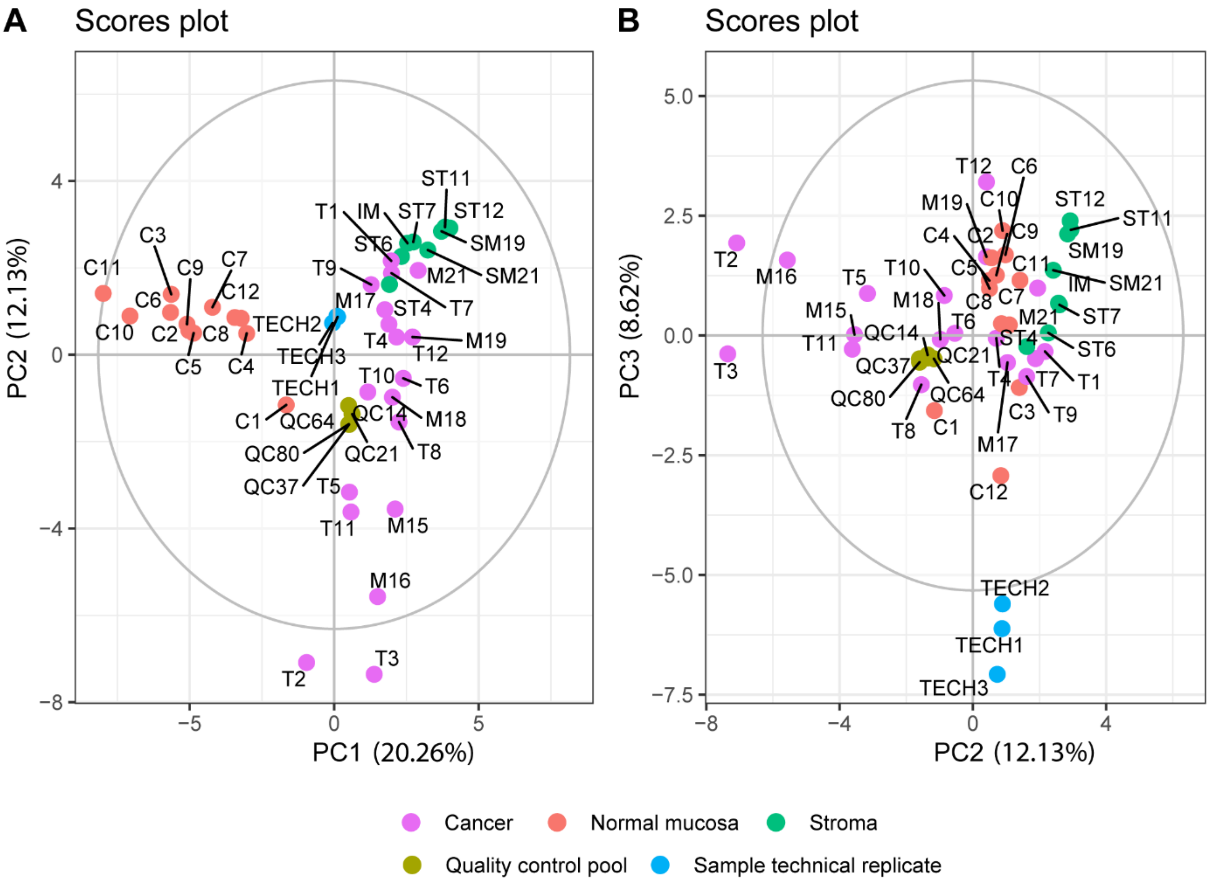

Figure S3. **Technical variation observed for the full workflow.** PC1 and PC2 score plot **A)** together with PC2 and PC3 score plot **B)** based on relative abundances of all *O*-glycans in all measured samples as well as technical (TECH1/2/3) replicates and QC pools (QC14/21/37/64/80) display close clustering of scores showing robustness of the method. The top three principal components explain 39.7 % of the variation within the data.

Figure S4

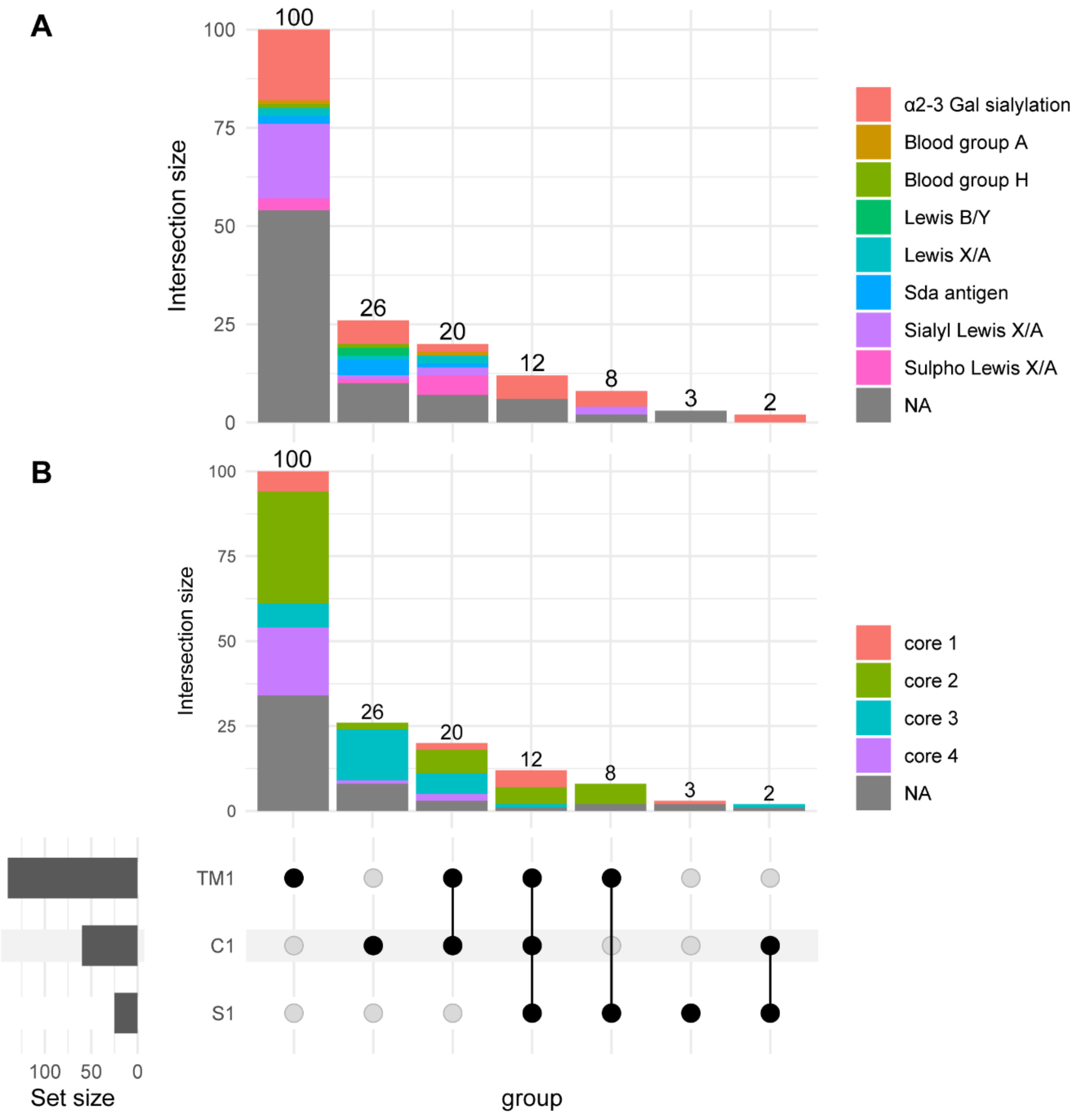

***Figure S4. O*-glycan specificity among cancer, tumor microenvironment controls and healthy mucosa controls.** Upset plot depicting the number of glycans detected specifically in each group (TM = adenocarcinoma, S = microenvironment controls (stroma, immune infiltrate), C = control normal mucosa) and their intersections indicating the number of shared glycans between the groups. Bars are colored by **A)** terminal epitopes or **B)** core structures present on the *O*-glycans. Counts are displayed on top of each bar. The NAs indicate glycans with either unassigned structures, or assigned structures that did not contain a specific epitope on the list.

Figure S5

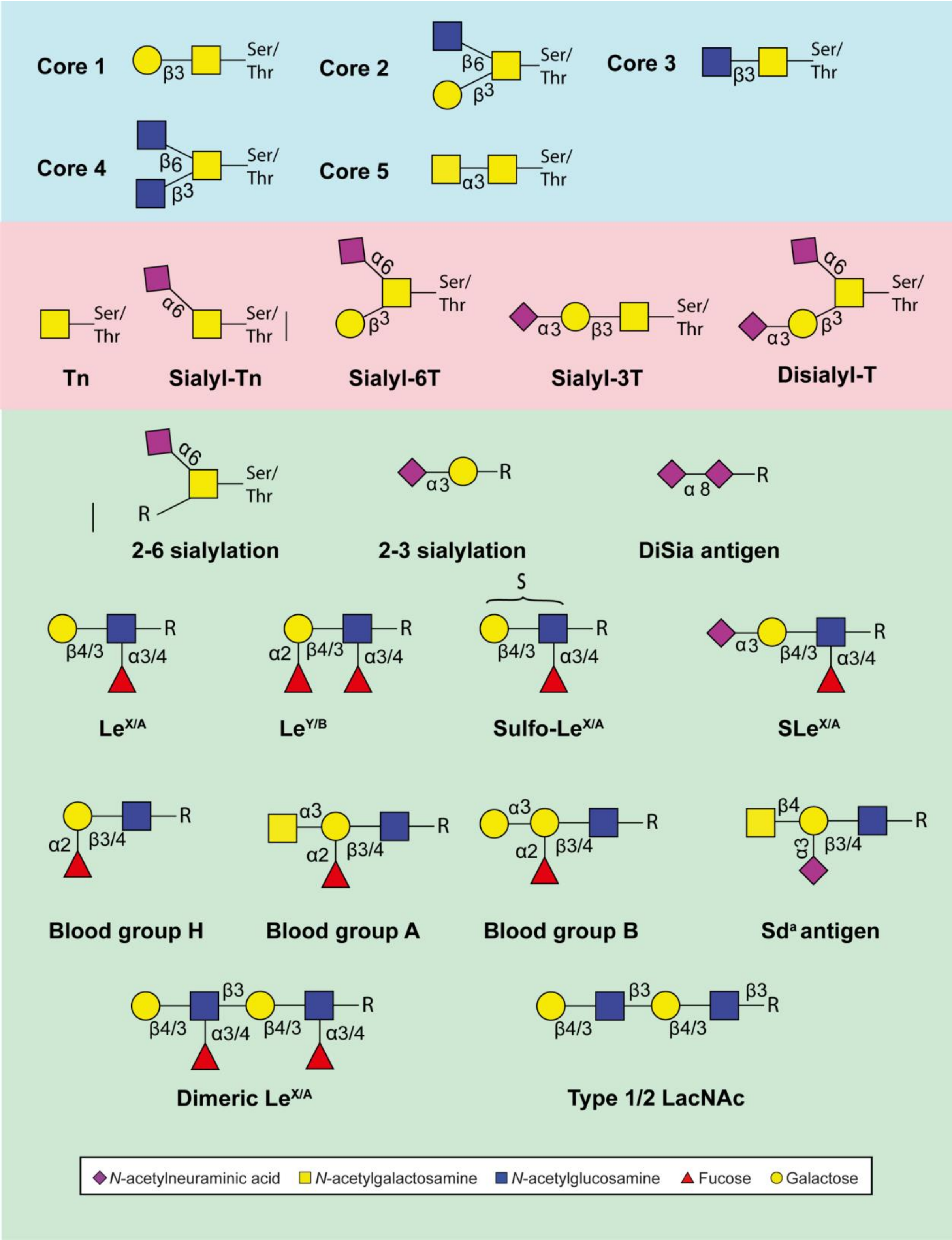

Figure S5. Commonly observed glycan cores, truncated glycan structures, terminal epitopes and extensions. *The figure depicts the five most commonly observed mucin type O-glycan core structures (1-5; in blue), commonly observed truncated glycans (pink), sialylation types, Lewis type antigens, blood group antigens (ABH) and core extensions (green).*

Figure S6

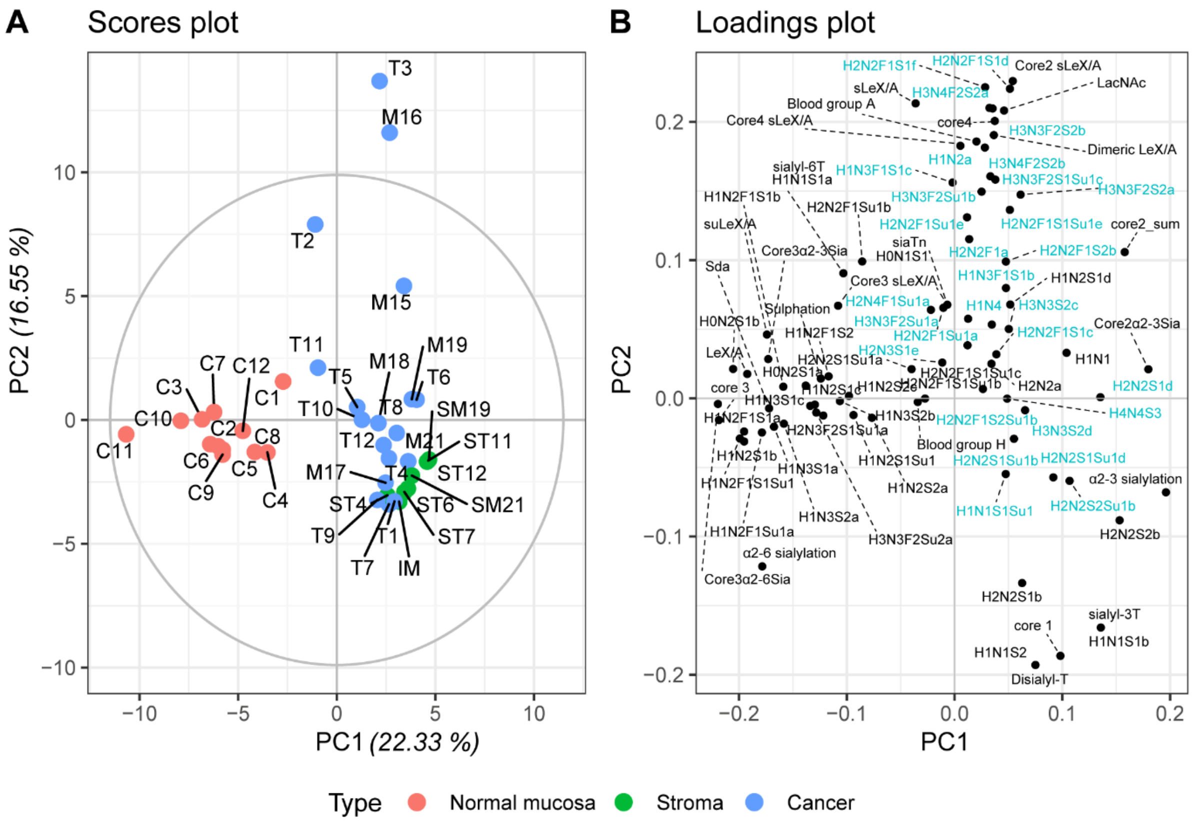

***Figure S6. O*-glycomic signatures separate cancer from healthy colon mucosa**. PCA model based on relative abundances (%) of individual glycans as well as calculated structural *O*-glycan features. Separation between cancer, microenvironment controls and healthy mucosa is illustrated in **a)** the PCA score plot of PC1 against PC2. **b)** The PCA loading plot displays the variables that drive the separation in the PCA model. The TACAs driving the separation of cancer and control samples are depicted in blue. The top two principal components explain 40.5% of the variation within the data.

Figure S7

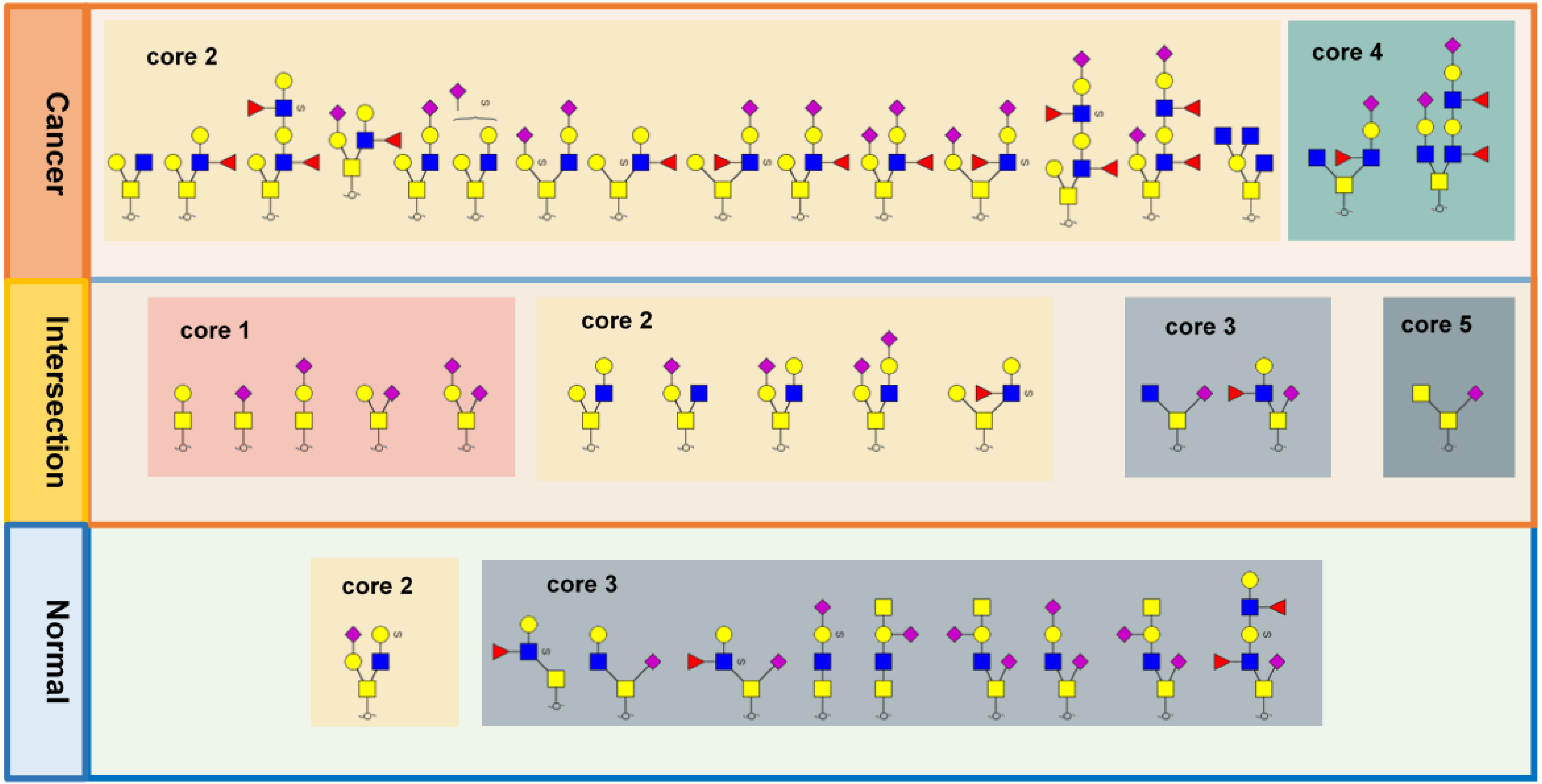

Figure S7. Region specific O-glycan structures. *O*-glycans are depicted when they are expressed in more than 15% of the cancer epithelia-TACAs (top section, orange), 15% of the normal colon mucosa (bottom section, blue), and selected structures expressed in both regions (intersection; yellow). Blue square: *N*-acetylglucosamine, green circle: mannose, yellow circle: galactose, red triangle: deoxyhexose, pink diamond: *N*-acetylneuraminic acid.

Figure S8

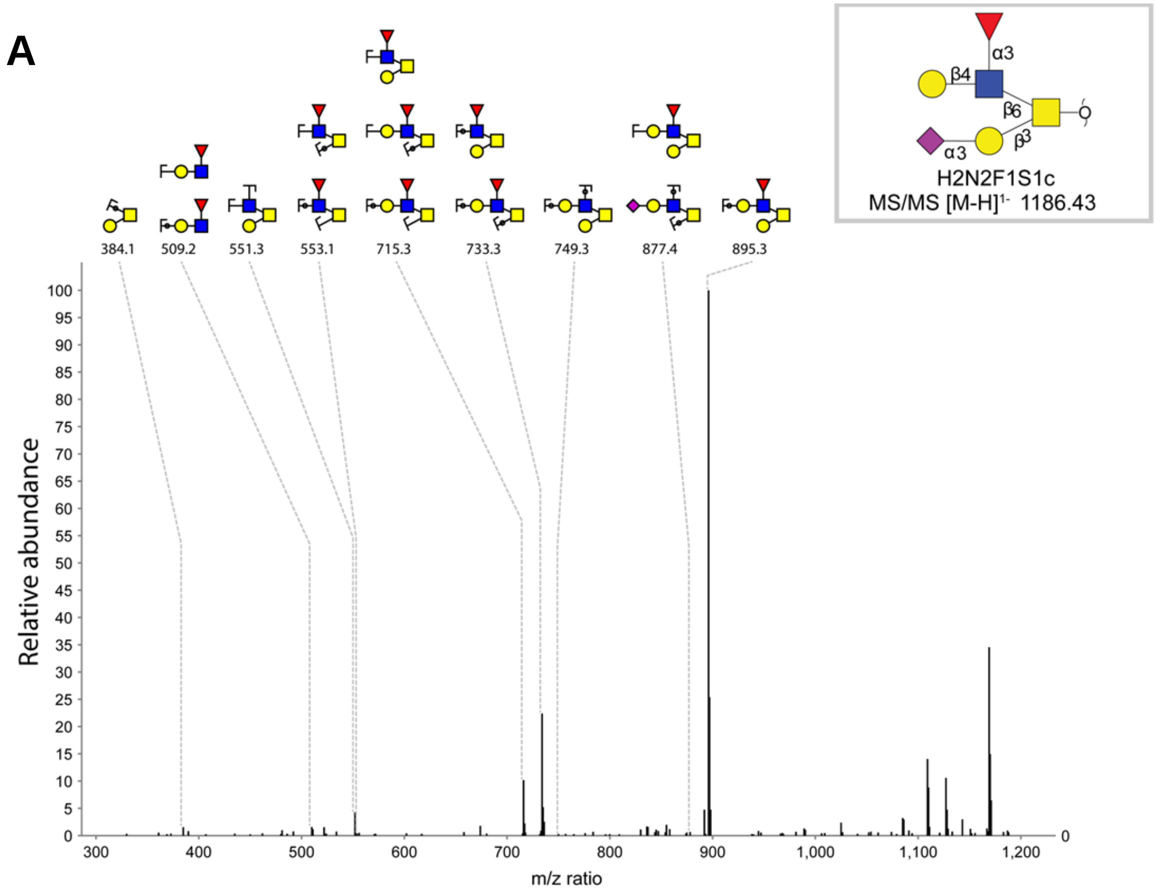

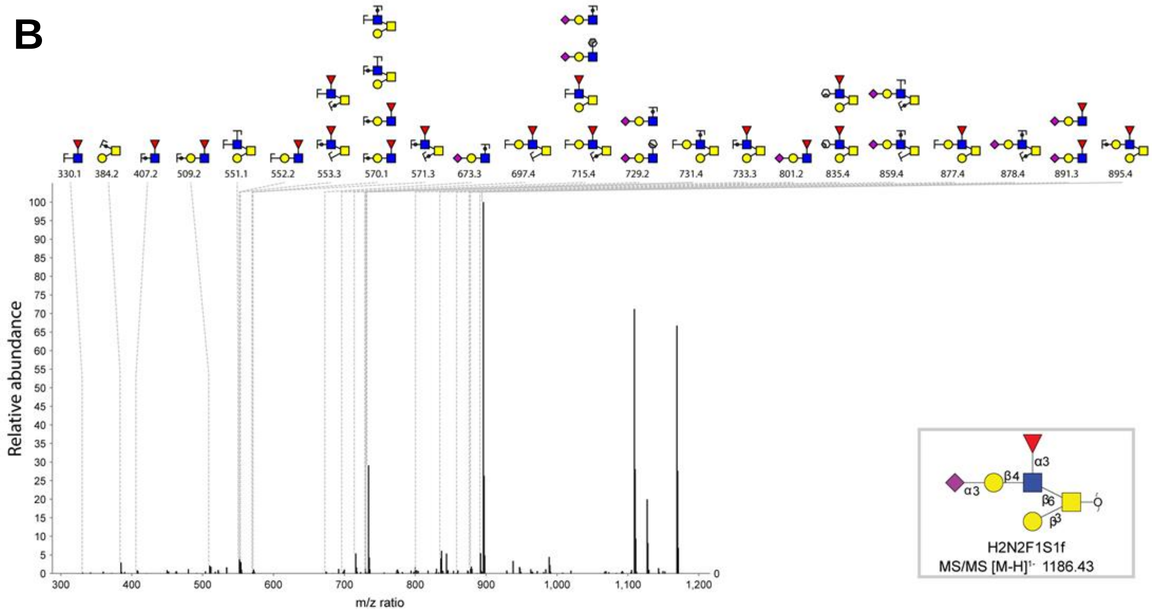

Figure S8. MS/MS spectra of the selected TACAs: **a)** *O*-glycan with composition H2N2F1S1c; **b)** *O*-glycan with composition H2N2F1S1f. Blue square: *N*-acetylglucosamine, green circle: mannose, yellow circle: galactose, red triangle: deoxyhexose, pink diamond: *N*-acetylneuraminic acid. Detailed peaklists and annotations of MS/MS spectra for each structure can be found in the supplied Glycoworkbench files.

C

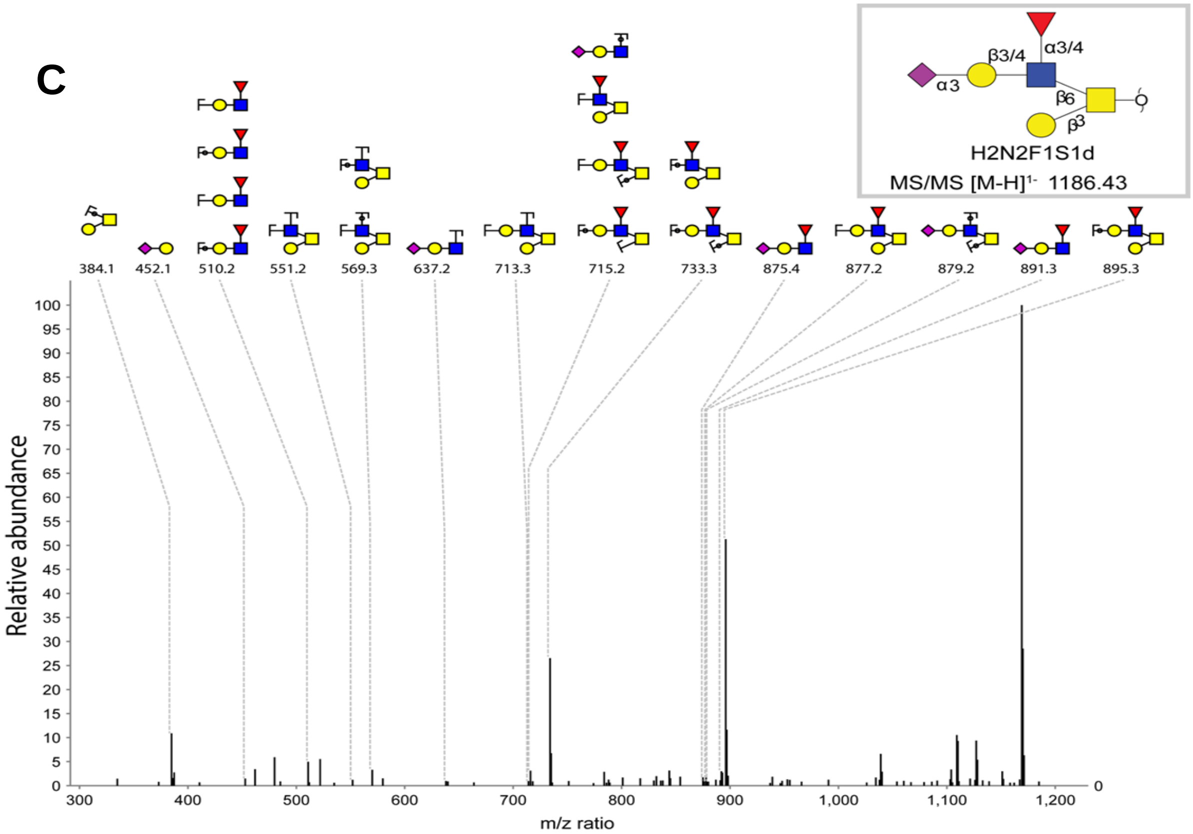

D

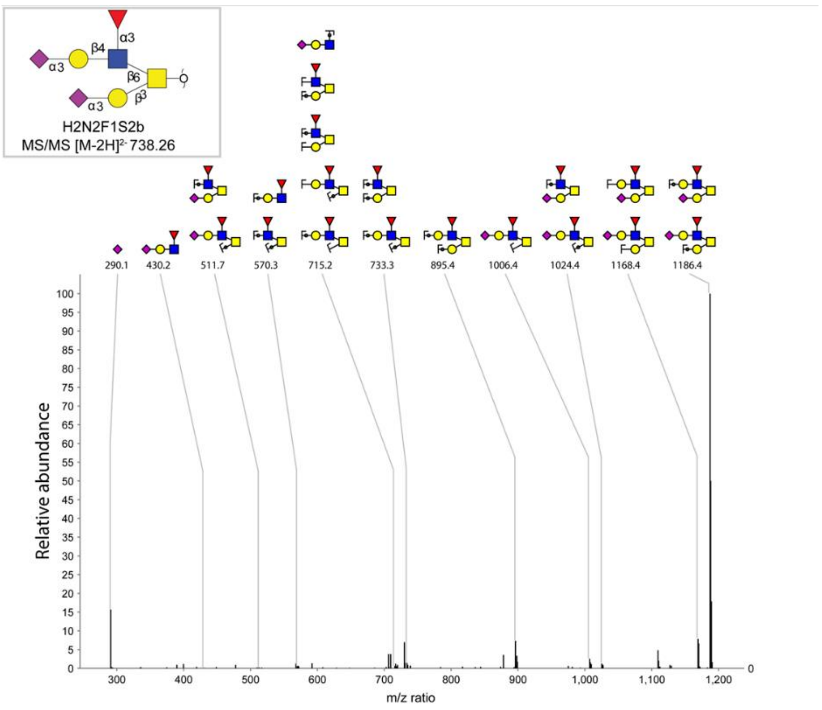

Figure S8 (continued). MS/MS spectra of the selected TACAs: **c)** *O*-glycan with composition H2N2F1S1d; **d)** *O*-glycan with composition H2N2F1S2b. Blue square: *N*-acetylglucosamine, green circle: mannose, yellow circle: galactose, red triangle: deoxyhexose, pink diamond: *N*-

acetylneuraminic acid. Detailed peaklists and annotations of MS/MS spectra for each structure can be found in the supplied Glycoworkbench files.

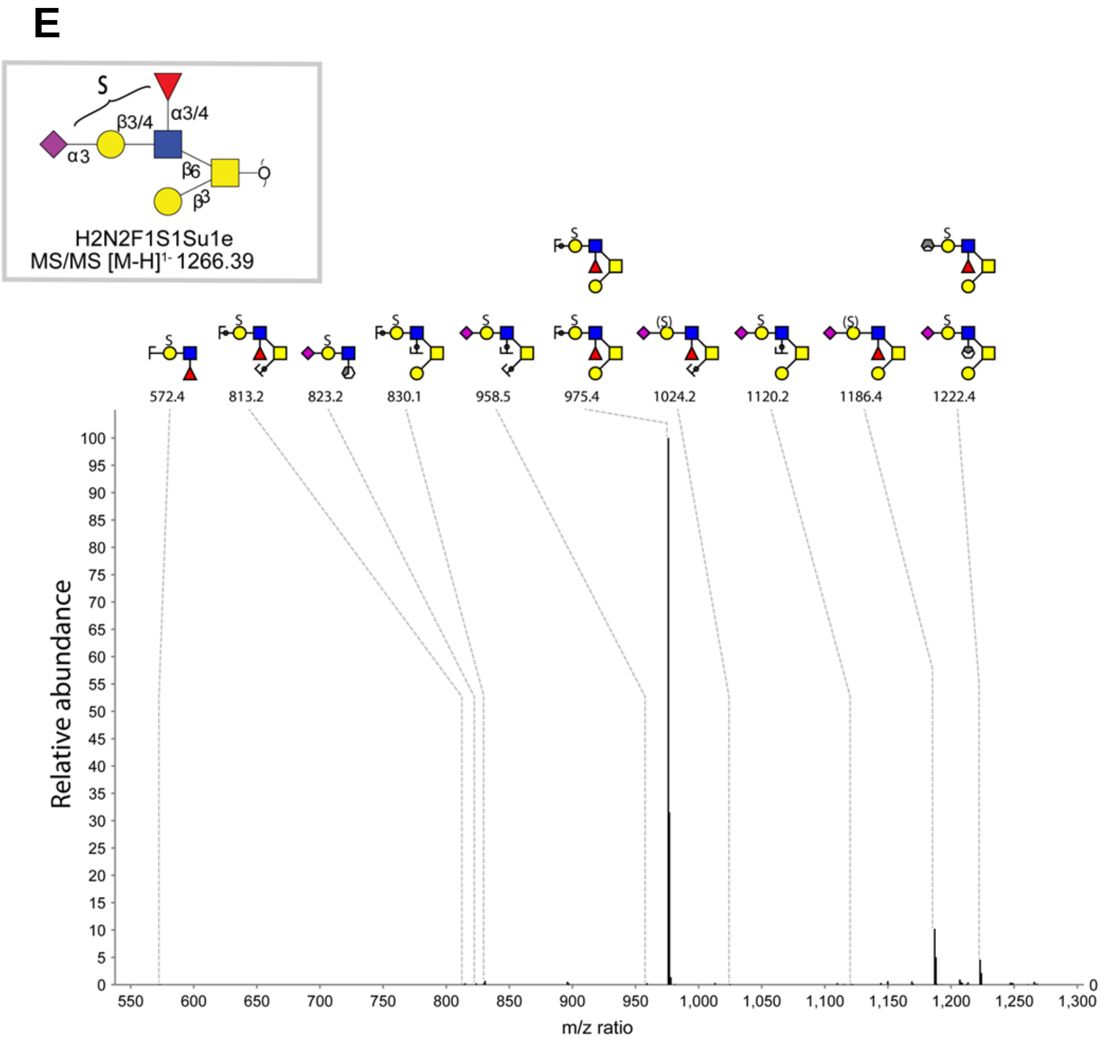

Figure S8 (continued). MS/MS spectra of the selected TACAs: **e)** *O*-glycan with composition H2N2F1S1Su1e. Blue square: *N*-acetylglucosamine, green circle: mannose, yellow circle: galactose, red triangle: deoxyhexose, pink diamond: *N*-acetylneuraminic acid. Detailed peaklists and annotations of MS/MS spectra for each structure can be found in the supplied Glycoworkbench files.

F

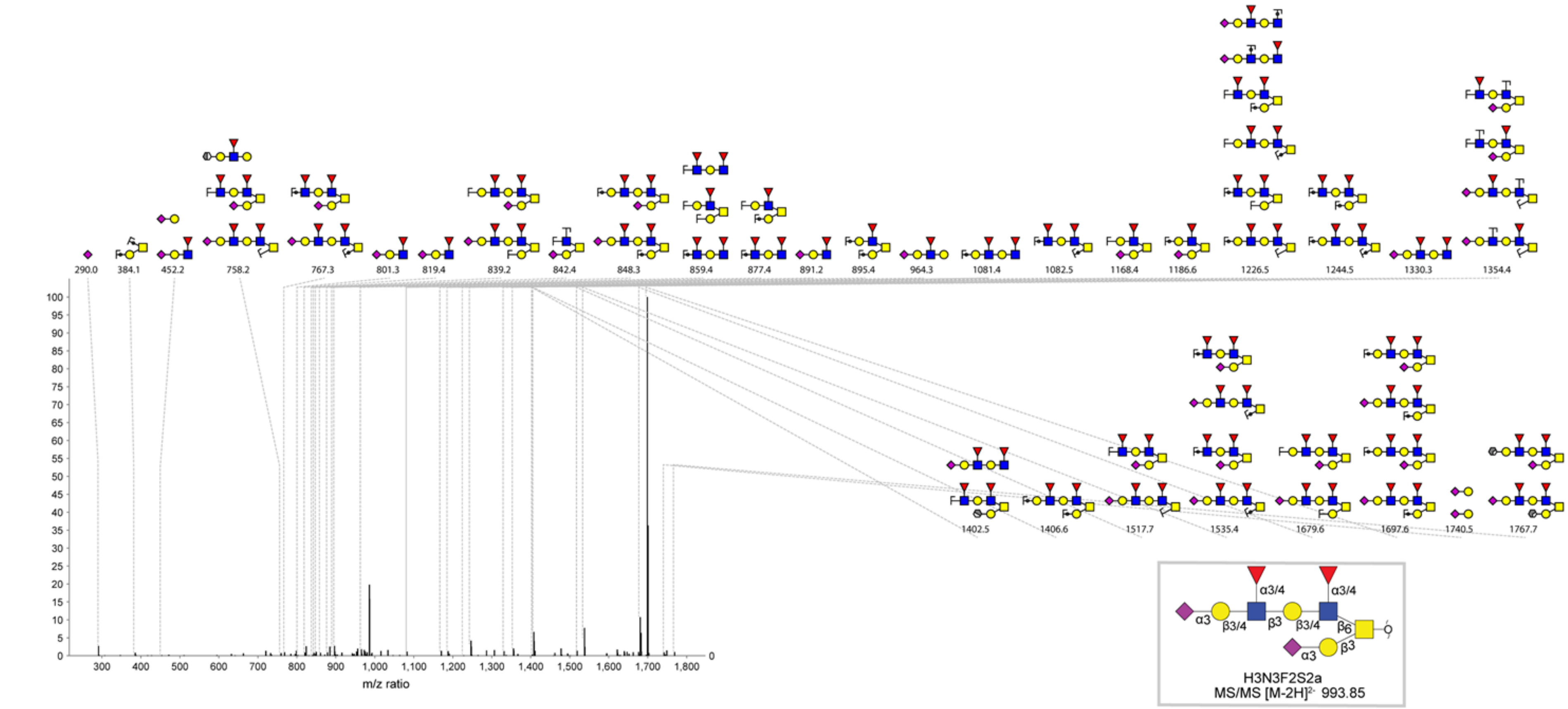

Figure S8 (continued). MS/MS spectra of the selected TACAs: **f)** *O*-glycan with composition H3N3F2S2a. Blue square: *N*-acetylglucosamine, green circle: mannose, yellow circle: galactose, red triangle: deoxyhexose, pink diamond: *N*-acetylneuraminic acid. Detailed peaklists and annotations of MS/MS spectra for each structure can be found in the supplied Glycoworkbench files.

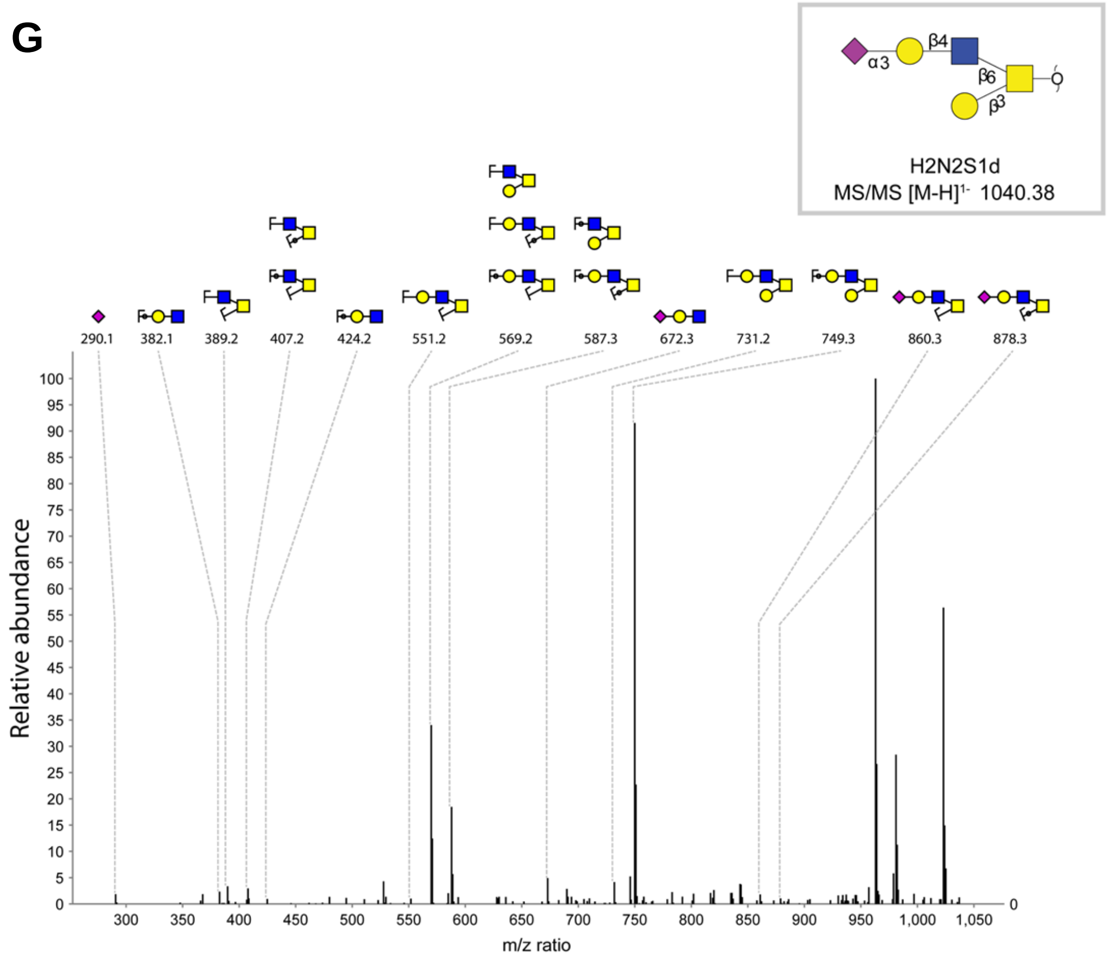

Figure S8 (continued). MS/MS spectra of the selected TACAs: **g)** *O*-glycan with composition H2N2S1d. Blue square: *N*-acetylglucosamine, green circle: mannose, yellow circle: galactose, red triangle: deoxyhexose, pink diamond: *N*-acetylneuraminic acid. Detailed peaklists and annotations of MS/MS spectra for each structure can be found in the supplied Glycoworkbench files.

Figure S9

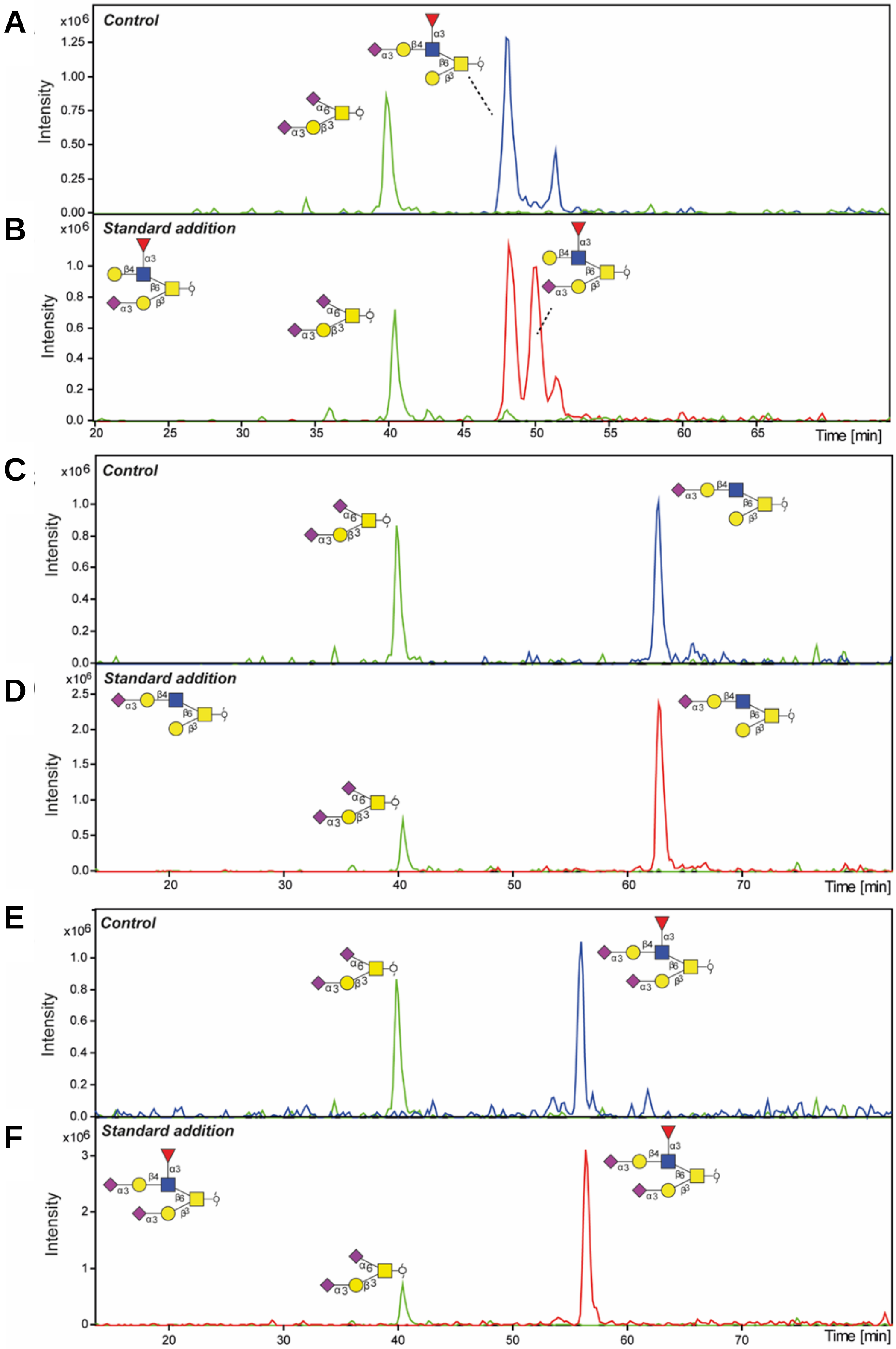

Figure S9. Standard addition. Chemically synthesized standards were added to the samples to confirm the glycan structures. For all panels **(A-F)** the green trace illustrates the EIC of control *O*-glycan peak (*m/z* = 966.3), **A, C, E)** the blue trace the EIC of the unknown peak and **B,D,F)** the red trace of the EIC of the spiked standard. Blue square: *N*-acetylglucosamine, green circle: mannose, yellow circle: galactose, red triangle: deoxyhexose, pink diamond: *N*-acetylneuraminic acid.

Figure S10

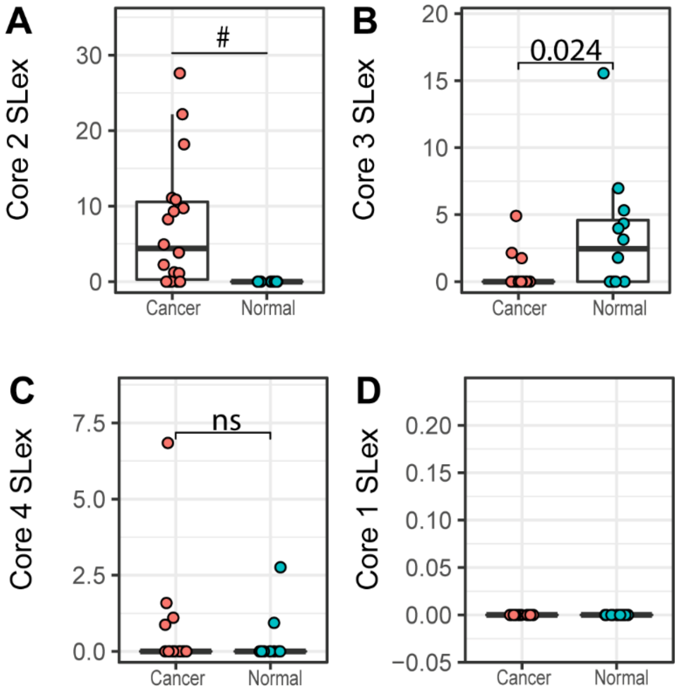

Figure S10. Expression of sLe$^{X/A}$ epitopes on different O-glycan cores. **A)** An upregulation is found in cancer of core 2 *O*-glycans that show sLe$^{X/A}$ expression, while **B)** a downregulation is found in cancer for sLe$^{X/A}$ epitopes carried by core 3 *O*-glycans. **C)** A limited subset of both cancer and normal colon mucosa samples express sLe$^{X/A}$ epitopes carried by core 4 with no significant difference. **D)** Core 1 *O*-glycans show no expression of sLe$^{X/A}$ epitopes in both cancer and normal mucosa.

Figure S11

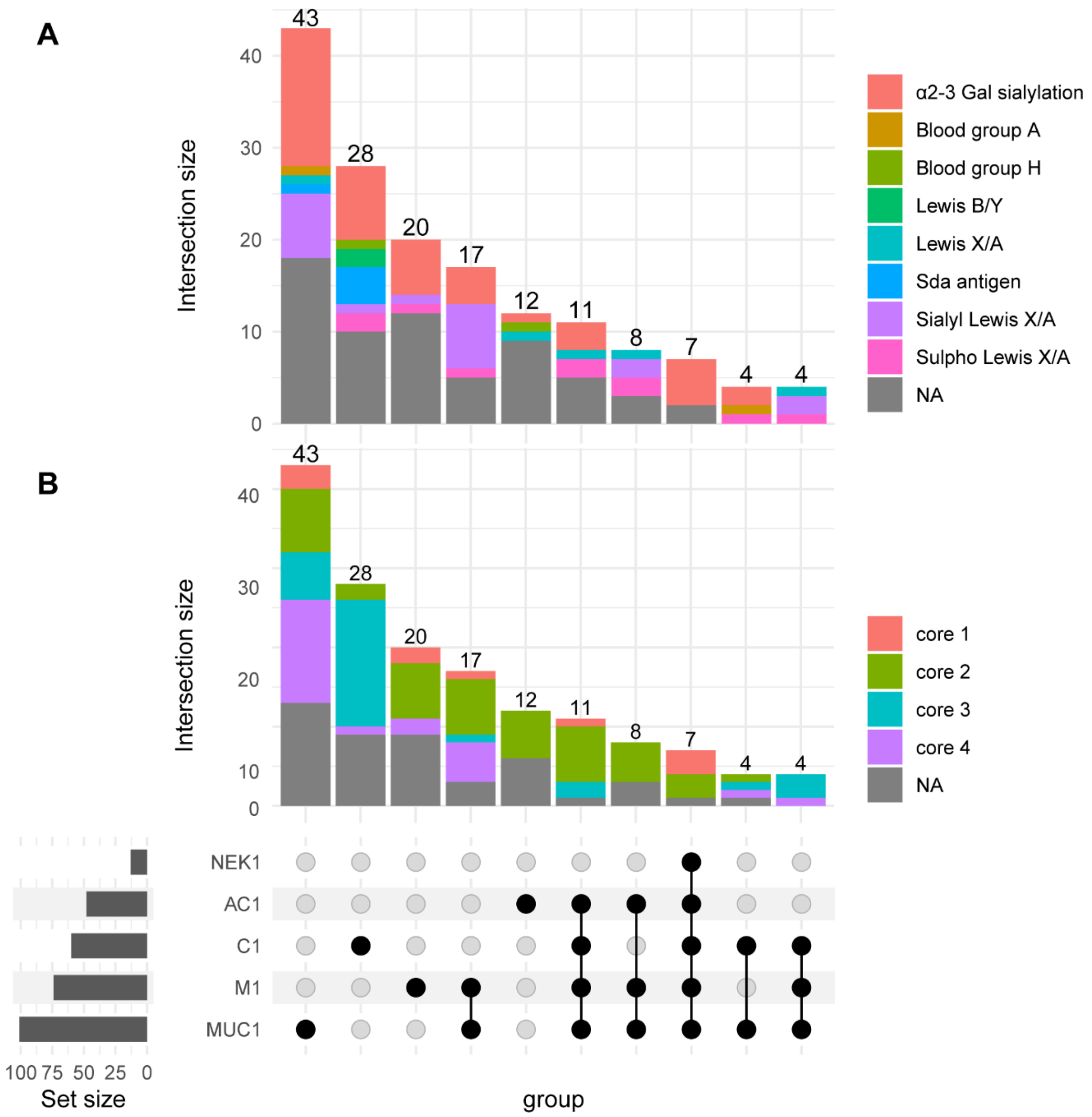

***Figure S11*. *O*-glycan specificity among different types of cancer and healthy mucosa controls.** Upset plot depicting the number of *O*-glycans detected specifically in each group (AC = adenocarcinoma, MUC = mucinous adenocarcinoma, NEK = neuroendocrine carcinoma, M = metastasis carcinoma, C = control normal mucosa) and their intersections indicating the number of shared *O*-glycans between the groups. Bars are coloured by **A)** terminal epitopes or **B)** core structures present on the *O*-glycans. Counts are displayed on top of each bar. The NAs indicate glycans with either unassigned structures, or assigned structures that did not contain a specific epitope on the list.

Figure S12

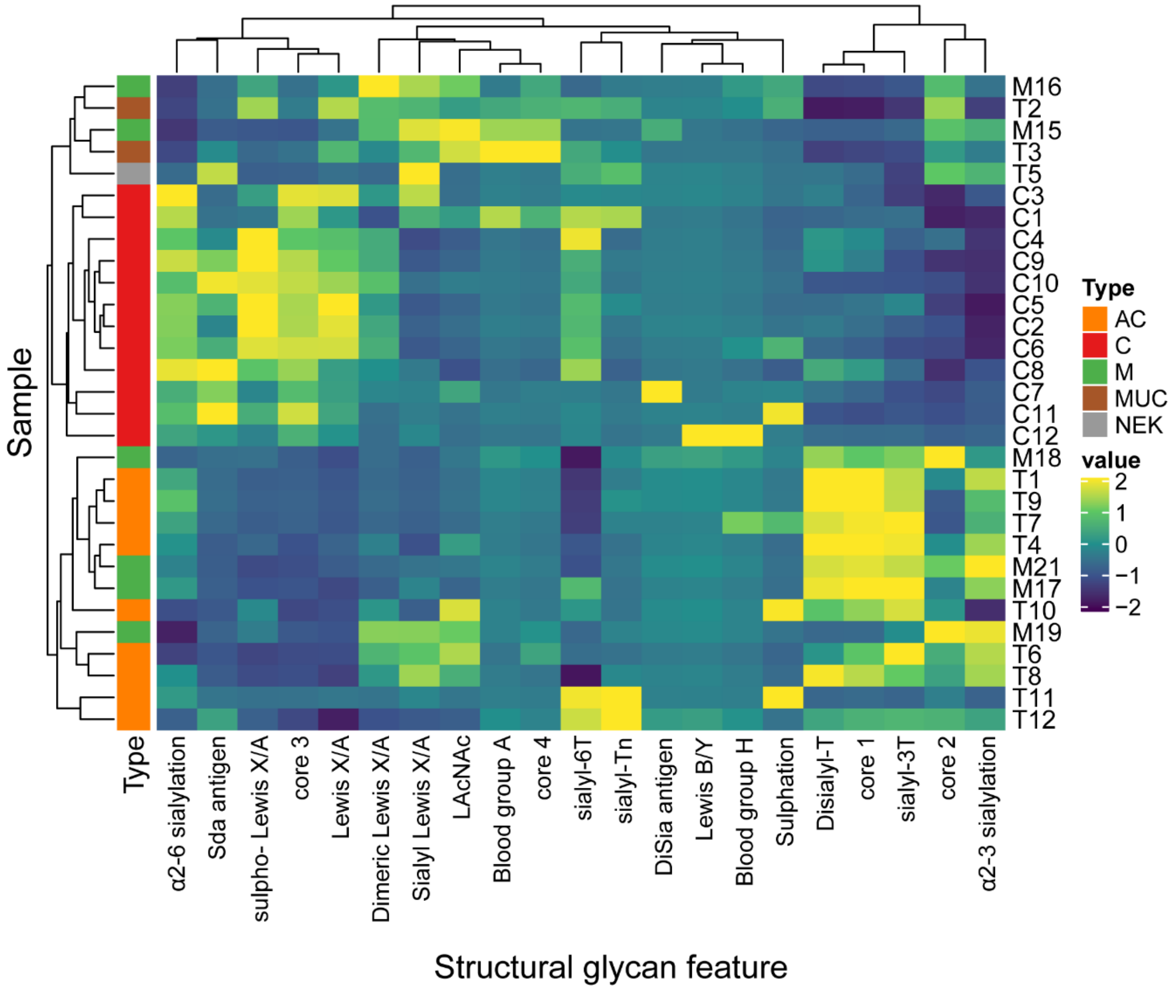

Figure S12. Hierarchical clustering based on structural O-glycan features. The graph illustrates the clustering of samples based on calculated structural *O*-glycan features shared between individual *O*-glycan structures. AC = adenocarcinoma, MUC = mucinous adenocarcinoma, NEK = neuroendocrine carcinoma, M = metastasis carcinoma, C = control normal mucosa.

Figure S13

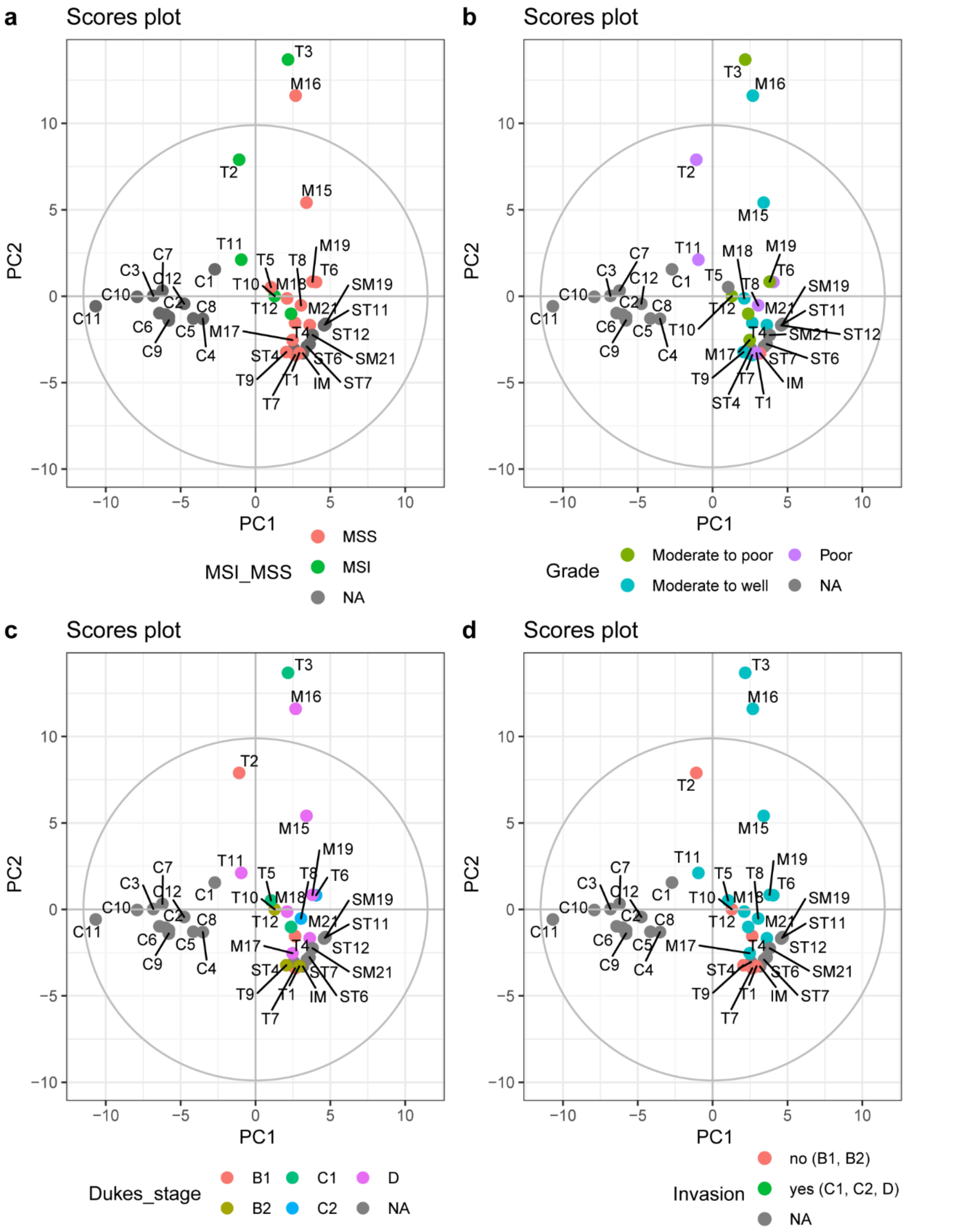

Figure S13. Association of glycomic signatures with cancer characteristics. PCA model based on relative abundances (%) of individual *O*-glycans as well as calculated structural *O*-glycan features and clustering based on **A)** microsatellite instability, ***B*)** differentiation grade, **C)** Dukes stage and **D)** Invasion to lymph node or distant organ. NA: not assigned.

Figure S14

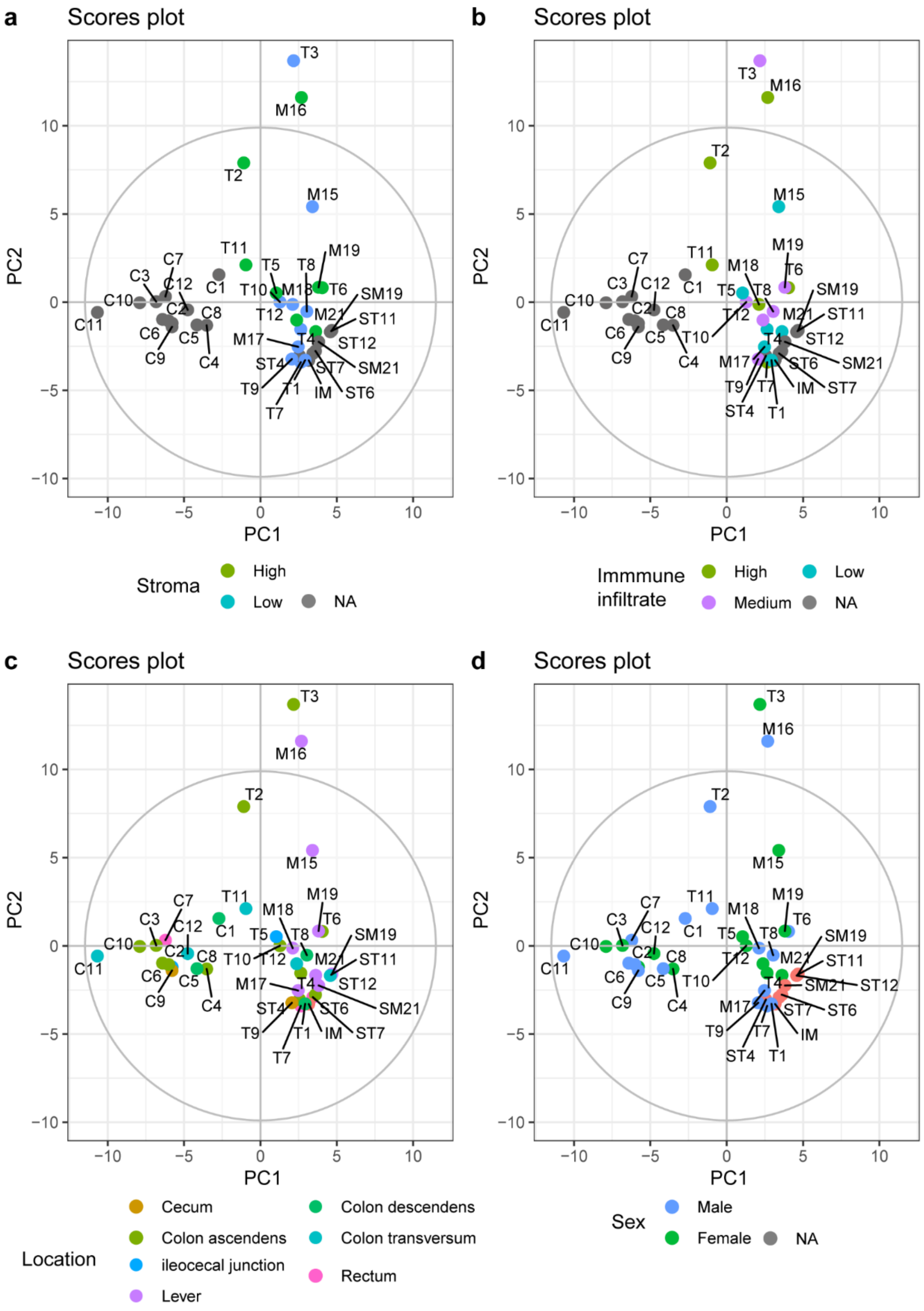

Figure S14. Association of glycomics signatures with cancer characteristics. PCA model based on relative abundances (%) of individual *O*-glycans as well as calculated structural *O*-glycan features and clustering based on **A)** stroma/tumor ratio, **B)** immune infiltration, **C)** cancer location and **D)** sex. NA: not assigned.

Figure S15

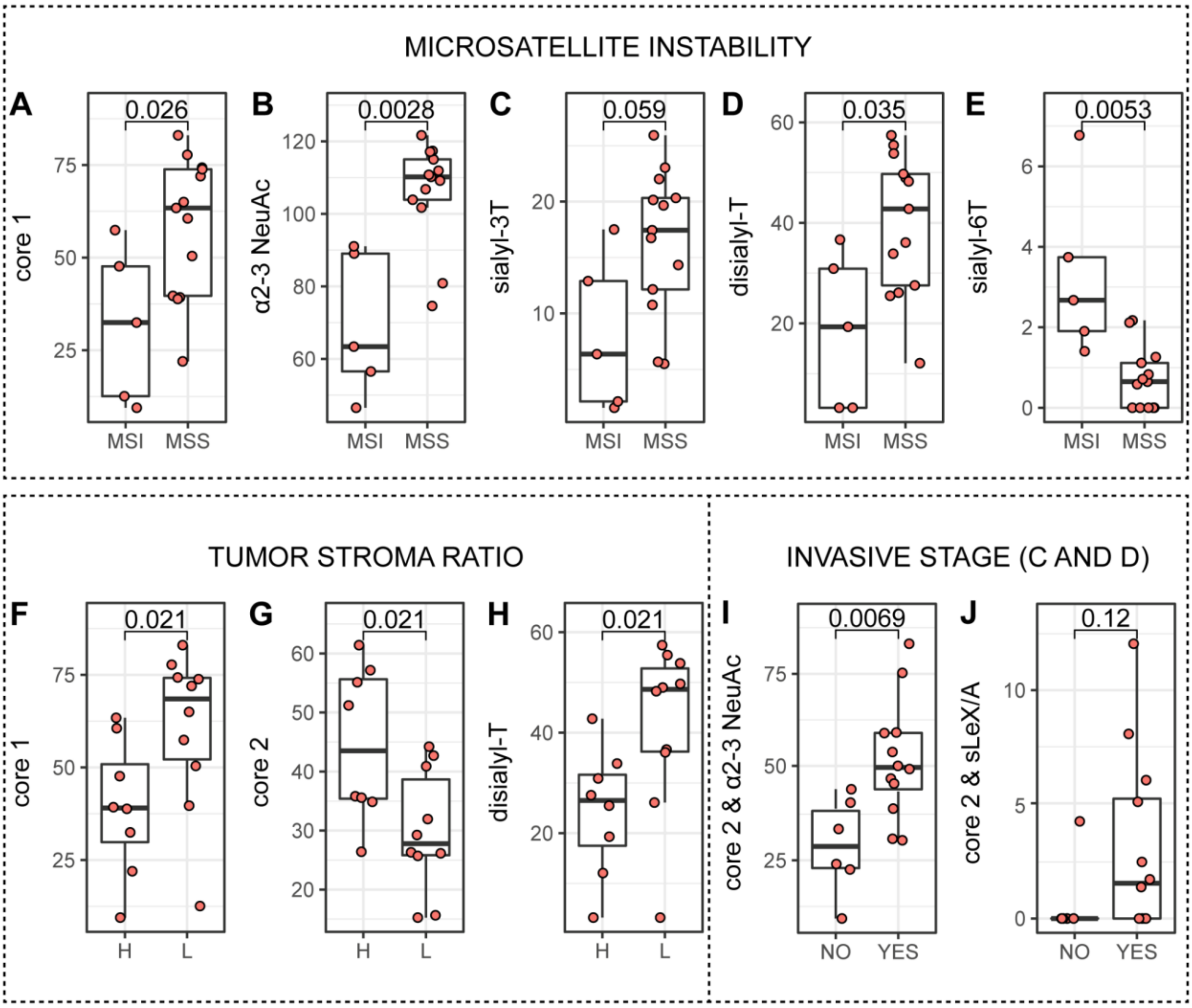

Figure S15. Structural glycan features that show difference between different types of cancers. Microsatellite instable cancers show downregulation of core 1 *O*-glycans and terminal α2-3 sialylation of Gal (**A, B**) including sialyl-3-T, disialyl-T (**C**, **D**), whereas they show an upregulation of sialyl-6-T with α2-6 linked sialic acid to the core GalNAc (**E**). Tumor-stroma ratio high cancers show downregulation of core 1 *O*-glycans including disialyl-T antigen (**F, H**), and an upregulation of core 2 structures (**G**). Cancers with invasion show upregulation of α2-3 sialylation of Gal and terminal sLe$^{X/A}$ antigen related to core 2 *O*-glycans (**I, J**), Differences between groups were tested using Wilcoxon-Mann-Whitney non-parametric statistical test.

Figure S16

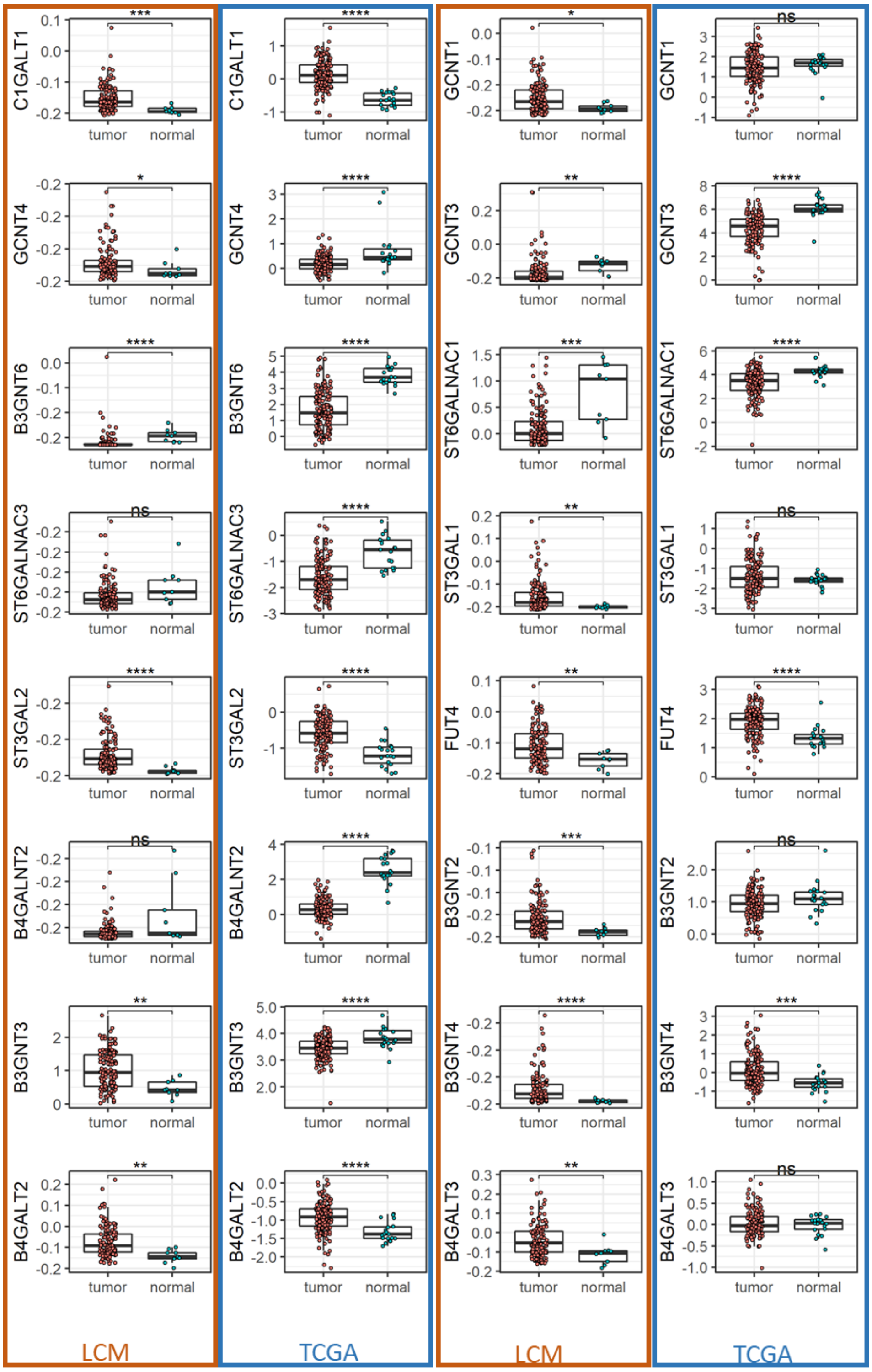

Figure S16. **Comparison of gene expression of glycosyltransferases in CRC and normal mucosa**. Statistically significant differences in expression in CRC compared to normal colon mucosa are observed for glycosyltransferases involved in the biosynthesis of observed *O*-glycans. The panel outlined in orange illustrates data from microdissected CRC tissues (LCM), whereas the panel outlined in blue contains data from TCGA (The Cancer Genome Atlas) dataset, without enrichment for cancer or normal epithelial cells. Only glycosyltransferase expression that showed significant differences between cancer and normal colon mucosa in either the LCM or TCGA dataset are shown.
